# Unraveling autophagy-metabolism crosstalk in cancer: Molecular insights and therapeutic strategies

**DOI:** 10.7150/thno.132362

**Published:** 2026-04-23

**Authors:** Haoyou Wang, Qianqian Yang, Yingying Lu, Cheng Du, Wei Wang, Lan Zhang

**Affiliations:** 1 Department of Thoracic Surgery, Liaoning Cancer Hospital & Institute, Cancer Hospital of China Medical University, Shenyang, 110042, China.; 2 Department of Thoracic Surgery, Cancer Hospital of Dalian University of Technology, Liaoning Cancer Hospital & Institute, Shenyang, 110042, China.; 3 Sichuan Engineering Research Center for Biomimetic Synthesis of Natural Drugs, School of Life Science and Engineering, Southwest Jiaotong University, Chengdu 610031, China.; 4 Department of Oncology, General Hospital of Northern Theater Command, Shenyang, 110316, China.

**Keywords:** autophagy, metabolism, metabolic reprogramming, cancer, small-molecule drugs

## Abstract

Autophagy is a catabolic process essential for the degradation and recycling of damaged proteins and organelles, thereby contributing to the maintenance of cellular homeostasis and the integrity of the intracellular environment. Although autophagy serves protective physiological functions, its involvement in various diseases, particularly cancer, is complex and context-dependent. In the context of tumor development, autophagy plays two distinct roles. During the early stages of tumorigenesis, it functions as a tumor suppressor by preserving genomic stability. In later stages, however, it promotes tumor growth, supports the survival of cancer cells, and contributes to therapeutic resistance. Cancer cells are known to change their metabolic processes to support growth and division. Autophagy and metabolism work together, enabling cells to utilize both external and internal resources to generate energy and synthesize new molecules. This interaction is especially important in the stressful environment of tumors, like when there's not enough food or oxygen. In these situations, autophagy helps the tumor adapt metabolically and grow by breaking down and reusing parts inside the cell. In this review, we systematically examine the role of autophagy as a key regulator that coordinates diverse metabolic programs in cancer cells. We focus on central metabolic pathways, including glycolysis, lipid metabolism, and amino acid metabolism, as well as emerging regulatory networks involving nucleotide metabolism and mitochondrial metabolism. Importantly, we highlight how these metabolic pathways are dynamically integrated through autophagy to facilitate tumor adaptation, support metabolic plasticity, and drive therapeutic resistance.

## 1. Introduction

Autophagy is a cellular self-digestion system that removes foreign substances and damaged cytoplasmic components, such as organelles and protein aggregates. This mechanism was first reported in human liver cells by Ashford and Potten in 1962, marking the first documented observation of the process 1. Although early research primarily described autophagy as a clearance mechanism for maintaining intracellular homeostasis, recent studies have shown that autophagy also plays a key role in coordinating metabolic adaptation under stressful conditions. In particular, starvation-induced autophagy enables cells to recycle intracellular components and reallocate metabolic substrates, thereby maintaining mitochondrial metabolism and energy balance 2. Importantly, a growing body of evidence suggests that the function of autophagy is not limited to simple cytoplasmic clearance, but rather that it acts as a dynamic regulator of metabolic plasticity in both normal physiological and pathological states. However, the role of autophagy in carcinogenesis is context-dependent and often paradoxical 3. In the early stages of tumour development, basal autophagy contributes to genomic stability by removing damaged mitochondria and aggregated proteins, thereby suppressing oxidative stress and chronic inflammation 4, 5. However, many cancer cells use autophagy, the body's own recycling mechanism, to strengthen their immune systems and increase their survival in the face of hypoxia and food restriction once tumors have developed. Autophagy may alleviate the metabolic stress caused by therapeutic pressure 6. It should be noted that this dichotomy between anti-tumor and tumor-promoting processes is not universal. Rather, it varies significantly depending on the tumor genotype, tissue of origin, and microenvironment. These complexities highlight the ongoing debate in the field regarding whether autophagy should be inhibited or stimulated to achieve therapeutic benefits. Thus, the fact that cancer cells depend on autophagy more than healthy tissues suggests that there is a window of opportunity for treatment.

Cell turnover is a series of interconnected anabolic and catabolic reactions that collectively regulate energy balance and protein synthesis 7. A key characteristic of cancer cells is metabolic reprogramming, in which oncogenic signaling pathways reshape the metabolic network to support rapid cell proliferation and biomass accumulation 8. Importantly, recent studies have shown that this metabolic reprogramming is not only driven by oncogenes but is also closely coordinated with stress response pathways such as autophagy. Consequently, autophagy is increasingly viewed not merely as a degradation process but as a metabolic regulator that supports tumor growth by dynamically modulating the availability of intracellular nutrients. Autophagy recycles metabolic macromolecules such as glucose, free fatty acids, amino acids, and adenosine triphosphate (ATP) 9. Abnormal metabolism promotes uncontrolled cell proliferation, disrupts epigenetic regulation, and reshapes the tumor microenvironment (TME). Metabolic reprogramming, particularly under hypoxic conditions, enhances tumor survival and promotes metastasis through interactions with stromal cells 10. In the nutrient-deprived microenvironments characteristic of many aggressive cancers, autophagy is widely activated as a survival mechanism, enabling cancer cells to withstand metabolic stress. By recycling intracellular macromolecules, autophagy provides essential nutrients for maintaining mitochondrial function, redox balance, and the biosynthetic pathways required for cancer growth 11. Nevertheless, there remains intense debate over whether autophagy primarily promotes tumor growth or, conversely, increases metabolic vulnerability. Some studies suggest that excessive activation of autophagy may trigger metabolic collapse or autophagy-dependent cell death, indicating that the quantitative levels of autophagy and its dynamic changes are key factors determining its functional outcomes in cancer 12. In addition, by disrupting cancer's metabolic processes, targeting autophagy has gradually emerged as a potential therapeutic approach.

To comprehensively delineate the interplay between autophagy and cancer metabolism, this review not only focuses on canonical metabolic pathways, including glycolysis, lipid metabolism, and amino acid metabolism, but also integrates emerging dimensions such as nucleotide metabolism and mitochondrial metabolism. Rather than functioning as isolated processes, these metabolic pathways are highly interconnected and collectively form a dynamic metabolic network that is tightly coordinated by autophagy. We aim to elucidate how autophagy orchestrates this multi-layered metabolic reprogramming to sustain tumor growth, enable metabolic plasticity, and confer therapeutic resistance. Furthermore, we summarize recent advances in small-molecule agents and rational combination strategies targeting the autophagy-metabolism axis, providing a conceptual framework for future translational applications.

## 2. Autophagy and cancer

Autophagy is a highly conserved intracellular degradation process that maintains cellular homeostasis by recycling cytoplasmic components and clearing damaged organelles or misfolded proteins, particularly under conditions of cellular stress and aging 13. Autophagy encompasses several distinct pathways, including macroautophagy, microautophagy, and chaperone-mediated autophagy (CMA), which collectively maintain protein homeostasis and organelle quality 5, 14. Traditional descriptions of autophagy have primarily focused on the classic stages of autophagosome formation and lysosomal degradation. These three main forms of autophagy utilize different molecular mechanisms to transport targets to the lysosomes 15. Among these, macroautophagy has been studied most thoroughly; its process follows a standard sequence of events, including initiation, phagosome nucleation, membrane extension accompanied by cargo separation, and the fusion of the autophagosome with the lysosome, followed by the degradation of the enclosed contents 16. More than twenty essential autophagy proteins, which are expressed by autophagy-related genes (ATGs), aid in the autophagy process. These proteins encapsulate a cellular component within a double membrane to form what is known as an autophagosome 17. When triggered by nutrient deprivation or oxidative stress, the autophagy mechanism is activated, leading to the formation of a membrane-bound compartment. This structure continuously expands to engulf damaged proteins or organelles, then closes to form a double-membraned autophagosome 18. The autophagosome eventually fuses with a lysosome, where its contents are broken down; the resulting breakdown products are then recycled by the cell to maintain its energy balance and homeostasis 19, 20. Furthermore, current evidence suggests that the autophagy network is more robust than this heritable concept, and that many aspects of it are closely linked to cancer biology 21. Specific forms of autophagy, such as mitochondrial autophagy, lipid autophagy, and aggregate autophagy, may exert signaling pathway-specific effects on cellular metabolism 22, 23. Although this classical framework has been extensively described, it does not fully capture the functional diversity of autophagy in cancer. A growing body of evidence suggests that it is not merely a process of bulk degradation, but rather that selective autophagy plays a dominant role in shaping metabolic outcomes. Crucially, the extent to which these distinct autophagy programs contribute to tumor progression remains unclear, and this influence varies significantly depending on tumor type, stage, and microenvironmental conditions. Furthermore, studies indicate that many key ATG proteins also possess non-autophagic functions that influence signaling pathways and vesicular transport 24. Since autophagy can produce extracellular effects in the tumor microenvironment, the significance of its spatial regulation for cell-type-specific or organelle-restricted autophagy is becoming increasingly acknowledged. Given these complexities, the investigation of autophagy mechanisms within a metabolic context cannot be limited to a simple description of classical pathways. In particular, when interpreting metabolic phenotypes, it is essential to distinguish between selective and non-selective autophagy and to consider the autophagy-independent functions of ATG proteins. Therefore, it is crucial to identify which selective autophagy processes are directly involved in the metabolic demands of tumor growth and treatment resistance.

**Figure 1:**
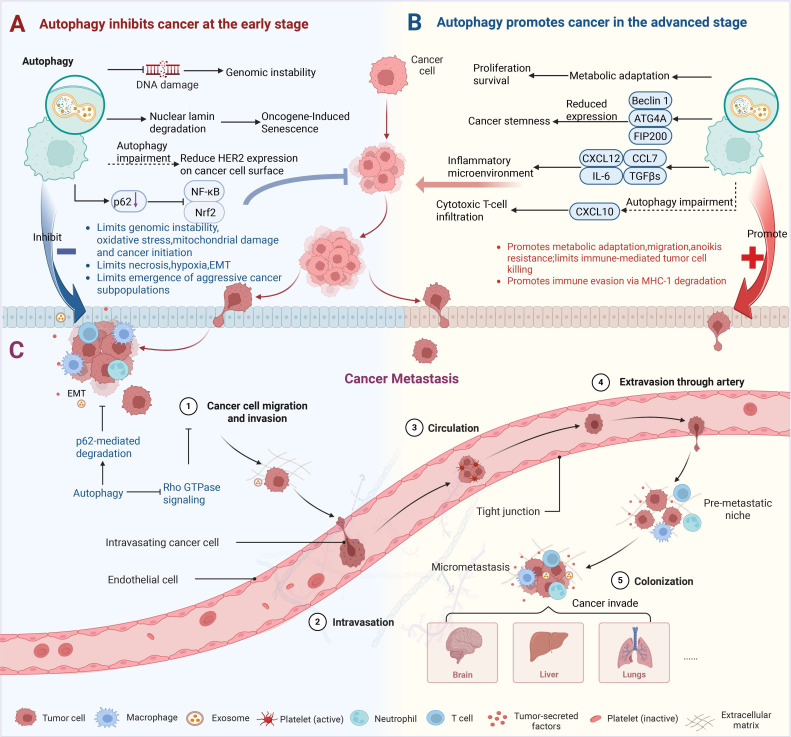
** The dual role of autophagy in cancer.** (A) Autophagy inhibits the occurrence of cancer by suppressing multiple pro-tumorigenic events, including cell survival, angiogenesis, inflammation, genomic instability, and HER2 expression on the cell surface. Additionally, autophagy induces senescence, which inhibits tumor formation. (B) Autophagy promotes the progression of cancer: once tumors form, autophagy drives progression by enhancing tumor cell proliferation, survival, invasion, growth during metastasis, and tumor stemness. (C) The stage-specific role of autophagy during metastasis progression. Preclinical evidence suggests that autophagy can both inhibit tumor growth and promote tumor progression at different stages of the metastatic cascade.

According to recent studies, autophagy is pathophysiologically involved in a number of human diseases, such as autoimmune disorders, cancer, and neurological conditions 25, 26. Autophagy plays a dual role in cancer development, and its specific manifestations vary depending on the type of cancer, its stage, or the patient's genetic background 27, 28. During the precancerous phase, autophagy is a vital mechanism of self-degradation that plays a key role in maintaining the homeostasis of cells and tissues. It eliminates mitochondria impaired by reactive oxygen species (ROS), counteracts oncolytic viruses by breaking them down, and helps maintain genomic stability 29. Furthermore, autophagy regulates oncogene-induced senescence by breaking down nuclear lamins 30. In addition to promoting the lysosomal degradation of pro-tumor factors, autophagy's tumor-suppressive effects can also be mediated by altering other transport pathways 31. A study indicates that human epidermal growth factor receptor 2 (HER2) expression on the surface of tumor cells is reduced when focal adhesion kinase family interacting protein of 200 kDa (FIP200) mediated autophagy is disrupted, which eliminates mammary tumorigenesis in MMTV-Neu 32, 33. Through these multifaceted functions, autophagy acts as a critical protective mechanism in precancerous cells 34. Another crucial tumor-suppressive mechanism of autophagy involves the selective degradation of the autophagy cargo receptor sequestosome 1 (SQSTM1/p62). When autophagy is compromised, p62 builds up and triggers the transcription factors nuclear factor kappa-B (NF-κB) and nuclear factor erythroid 2-related factor 2 (Nrf2) 35, 36 (Figure 1A). This mechanism promotes cell survival, angiogenesis, and inflammatory responses, thereby creating a microenvironment that supports tumor growth 37. Furthermore, degradative autophagy removes damaged mitochondria and inflammasome components, thereby negatively regulating inflammasome activation. Autophagic defects can lead to cellular damage, necrosis, chronic inflammation, and genetic instability. These factors can increase cancer incidence by altering the tumor microenvironment, increasing oxidative stress, and causing oncogenic mutations 38. In cells and tissues with impaired autophagy, the inability to clear damaged proteins and organelles leads to cellular dysfunction and cell death, which in turn triggers inflammation and ultimately creates an environment conducive to cancer development 39.

However, the protective function of autophagy creates a therapeutic paradox, as established cancer tumors exploit this process to accelerate their growth and withstand stress. In the advanced phases of tumor formation, autophagy transitions from its initial role as an anticancer process to a critical supporting mechanism that facilitates tumor survival, progression, and metastasis 33 (Figure 1B). Through various mechanisms, autophagy significantly enhances tumor development and metastasis. First, autophagy breaks down damaged or unnecessary intracellular components, giving tumor cells vital metabolic substrates (including fatty acids and amino acids) to sustain their growth and survival in the nutrient-poor tumor microenvironment 11. Beyond metabolic adaptation, autophagy also drives tumor progression through other mechanisms. For instance, autophagy is required for cancer stem-like properties, which are associated with tumor initiation and resistance to therapy. Depletion of Beclin1 or autophagy-related 4 homolog A (Atg4A) in breast cancer cell lines or genetic ablation of Fip200 in a mouse model of breast cancer compromises the maintenance of cancer stem cells 40-42. The impact of autophagy on tumor progression is also profoundly shaped by the TME, wherein its function is spatially and temporally regulated, exhibiting distinct, often opposing, roles during early and late tumorigenesis 6, 43. A key modulator of this crosstalk is the heterogeneous population of cancer-associated fibroblasts (CAFs) 44. Under this dynamic regulation, CAFs drive malignancy through paracrine signaling, secreting factors such as C-X-C motif chemokine ligand 12 (CXCL12), interleukin-6 (IL-6), chemokine (C-C motif) ligand 7 (CCL7), and transforming growth factor-β superfamily (TGFβs) to foster a pro-tumor inflammatory niche that enhances cancer cell survival, proliferation, stemness, and metastatic initiation 45. Moreover, the tumor-promoting function of autophagy can also be mediated by suppressing immune surveillance. For example, in the MMTV-PyMT mouse mammary tumor model, genetic ablation of Fip200 resulted in an autophagy defect that enhanced the production of the chemokine C-X-C motif chemokine ligand 10 (CXCL10) and increased the infiltration of cytotoxic T cells into the primary tumor 31.

One of the most distinctive characteristics of cancer is metastasis, which occurs when cancer cells spread from the original tumor and use the lymphatic and circulatory systems to enter and colonize distant organs 46, 47. Autophagy plays a complicated and situation-specific role in cancer metastasis. In the early stages, autophagy inhibits metastasis by hindering tumor necrosis and cell infiltration 48. By preventing pro-tumorigenic traits like proliferation, survival, migration, and invasion, therapeutic targeting of autophagy genes like Beclin1 and microtubule-associated protein 1 light chain 3 (MAP1LC3) offers a viable therapy approach for breast cancer 49. Furthermore, early-stage cancers are associated with decreased expression of Atg5, an essential autophagy regulator, which promotes the proliferation of cancer cells 50. An investigation further reveals that inhibiting mTOR signaling induces autophagy-dependent cell death, hence limiting metastatic dissemination in gastric cancer cells 51 (Figure 1C). However, in advanced metastatic diseases or conditions, autophagy facilitates cancer cell migration, enhances the colonization of dissociated cells, induces metastatic cells to enter a dormant state, and enables them to survive in new environments 52. One study has found that inhibiting autophagy inhibits the metastatic capabilities of hepatocellular carcinoma (HCC) cells by lowering their invasion, migration, and resistance to anoikis, therefore suppressing lung metastasis *in vivo* 53. After reaching the target organ colonization stage, cancer cells induce autophagy flux to counteract hypoxia and nutritional restrictions 54. A key mechanism through which autophagy may exert these pro-metastatic effects is the regulation of epithelial-mesenchymal transition (EMT) 55. By enhancing cell motility and invasiveness, EMT serves as a pivotal driver of tumor cell dissemination and, concomitantly, can mediate drug resistance in human cancers 56, 57. Autophagy has been identified as a crucial process allowing tumor adaptation and survival under therapeutic pressure, since drug resistance continues to be a significant obstacle in cancer treatment 58. In this light, autophagy has become a therapeutic target that shows promise for overcoming treatment resistance.

The dual role of autophagy in cancer is also one of the most controversial topics in this field. While the general paradigm of tumor-suppressive autophagy in early stages and tumor-promoting autophagy in advanced stages is widely accepted, emerging evidence challenges this simplistic view. A recent study reveals that alkb homolog 5 (ALKBH5) mediated autophagy inhibition drives the progression of ovarian cancer and melanoma, while ALKBH5-mediated autophagy activation exerts tumor-suppressive effects in colorectal and gastric cancers 59. This suggests that even in early stages, autophagy's tumor-suppressive function may depend on specific contexts. Furthermore, the degree to which autophagy-dependent cancers depend on their particular oncogenes varies significantly; for instance, autophagy is necessary for KRAS-induced lung cancer but not for BRAF-driven melanoma 60. Additionally, research indicates that autophagy's influence on metastasis is highly stage-specific, exhibiting both pro-metastatic and anti-metastatic activity based on the organ microenvironment and metastatic stage 52. These problems highlight the importance of context when developing autophagy-based therapeutic options. At the same time, autophagy's dual involvement in cancer and its function in cellular metabolism are closely intertwined 61. Autophagy is a critical metabolic process that helps cancer cells survive and proliferate by replenishing their metabolic resources. This process is triggered by various tumor-associated stressors, including hypoxia and nutrient deprivation 62. This metabolic adaptability is accomplished by the dynamic interaction of autophagy with major metabolic processes such as glycolysis, lipid metabolism, and amino acid metabolism, which will be discussed in greater detail in the following sections. In the following part, we'll look at how autophagy interacts with these critical metabolic pathways to drive tumor development and therapeutic resistance.

## 3. Autophagy and cancer metabolism: key metabolic pathways

**Figure 2:**
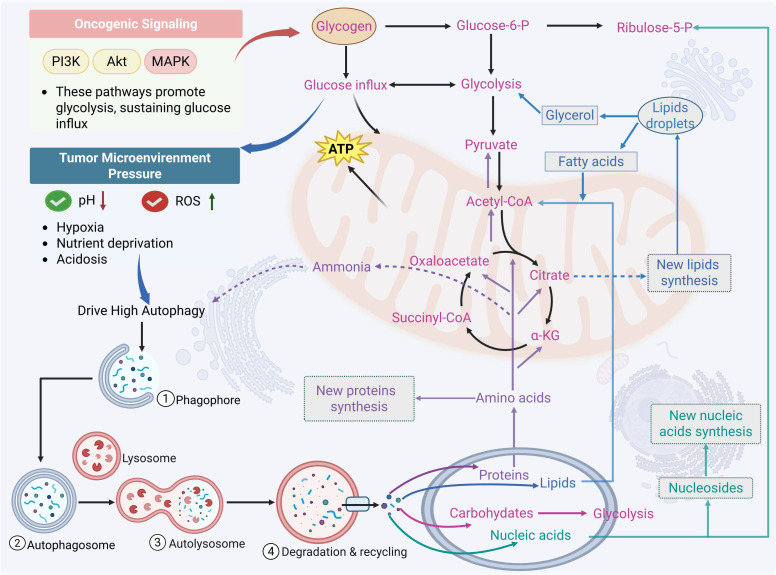
** An overview of the interaction between autophagy and cancer metabolism.** Abnormal gene regulation (including activation and inactivation of tumor suppressor genes) and harsh microenvironments (nutrient deficiency, hypoxia, acidosis, and interstitial pressure) trigger autophagy, leading to reprogramming of tumor metabolism. Glycolysis and pentose phosphate pathway (PPP) catabolism produce ATP and pyruvate for further metabolism in the tricarboxylic acid cycle (TCA) when glucose is released from glycogen granules by glycogenolysis or autophagy. Lipid droplets (LDs) or lipolytic or autophagic membranes produce fatty acids that are converted into acetyl-coenzyme A (acetyl-CoA), which supplies the TCA cycle and promotes the synthesis of ATP and citrate. Amino acids are frequently employed to create new proteins. Through the activity of aminotransferases, some amino acids can enter the tricarboxylic acid cycle route and produce citrate or oxaloacetate, which can be processed to produce ATP. They can also combine to form citric acid, which promotes lipid synthesis and membrane biogenesis. Ammonia, which is an autophagy activator, is produced during amino acid catabolism. Nucleosides are synthesized into new nucleic acids and catabolized via a combination of PPP and glycolysis. (The red, blue, purple, and green fonts, respectively, represent the metabolic processes related to sugar, fat, protein, and nucleotide.)

Autophagy is closely linked to metabolic reprogramming in cancer cells. A well-established hallmark of cancer is the reprogramming of metabolic pathways; this leads to the reshaping of key metabolic pathways that regulate the utilization of glucose, amino acids, and fats, thereby enabling tumor cells to proliferate and spread uncontrollably 63. To meet this increased metabolic demand, cancer cells use autophagy to convert catabolic products (such as amino acids and lipids) into energy and biosynthetic precursors. This interaction results in a vicious cycle that not only promotes tumor proliferation but also confers resistance to medicines 64 (Figure 2). Through coordinated regulation of several metabolic pathways, including glycolysis, lipid metabolism, amino acid metabolism, and emerging processes like nucleotide metabolism and mitochondrial metabolism, this section methodically investigates how autophagy promotes tumor survival and therapeutic resistance. Crucially, it is incorrect to think of these metabolic pathways as separate modules. Rather, a growing body of research indicates that autophagy serves as a central integrator that dynamically redistributes metabolic flux across linked pathways, allowing cancer cells to adjust to changing stress and nutritional conditions.

Tumors engage in bidirectional interactions with the body, actively scavenging nutrients and changing the distribution of nutrients throughout the body, while the availability of nutrients restricts their own growth and metabolism. Moreover, metabolites generated from tumors serve as signaling molecules that control gene expression and alter the activity of nearby stromal cells in addition to being sources of energy and biomass 10. This dynamic is further shaped by significant metabolic heterogeneity in tumor cells, which exhibit different metabolic adaptation phenotypes in response to changes in the external environment 65. For example, metastasis-associated in colon cancer 1 (MACC1) is significantly upregulated to facilitate the Warburg effect and ensure gastric cancer growth in glucose deprivation-induced metabolic stress 66. Under nutrient-deprived conditions, hepatic cancer cells activate the serine biosynthesis pathway to promote cancer progression by upregulating oncogene cellular myelocytomatosis oncogene (cMyc) 67. Depending on the stage of the disease's progression, autophagy has a different role in acute myeloid leukemia (AML). Reduced autophagy levels drive tumor transformation in the early stages of AML, while enhanced autophagy activity contributes to leukemic progression and poor treatment response in the advanced stages of this disease 68. By activating the adenosine monophosphate-activated protein kinase (AMPK)/Unc51-like kinase-1 (ULK1) pathway, lipocalin inhibits glycolysis and promotes autophagy in prostate cancer, reducing the growth and invasiveness of tumor cells 69. Additionally, the activation of ATG-dependent autophagy in prostate cancer (PCa) cells mediates resistance to glutamine deprivation, which gives the cells a survival advantage and acquired resistance to radiation 70. In multiple myeloma, autophagy supports tumor cell survival under hypoxia by enhancing glycolytic and mitochondrial activity. The ensuing upregulation of hexokinase 2 (HK2) further activates autophagy to enhance viability. Therefore, either through HK2 inhibition or direct blockade of autophagy effectively sensitizes myeloma cells to treatment 71.

In conclusion, autophagy is a key hub for metabolic adaptability in cancer, coordinating new metabolic processes like nucleotide turnover and mitochondrial quality control in addition to traditional pathways like glycolysis, lipid metabolism, and amino acid metabolism. Through this integrated regulation, autophagy maintains metabolic homeostasis and supports tumor progression under diverse stress conditions. Targeting autophagy or metabolic pathways not only helps reveal the molecular basis of drug resistance and tumor progression but also provides important theoretical support for the development of precision therapeutic strategies in multiple cancer types. Therefore, understanding how autophagy reprograms metabolism to support tumor growth would provide valuable insights to improve patient treatment. In this section, we review the regulation of tumor cell autophagy by various metabolic pathways and metabolites.

### 3.1 Interplay between autophagy and glycolysis metabolism

**Figure 3:**
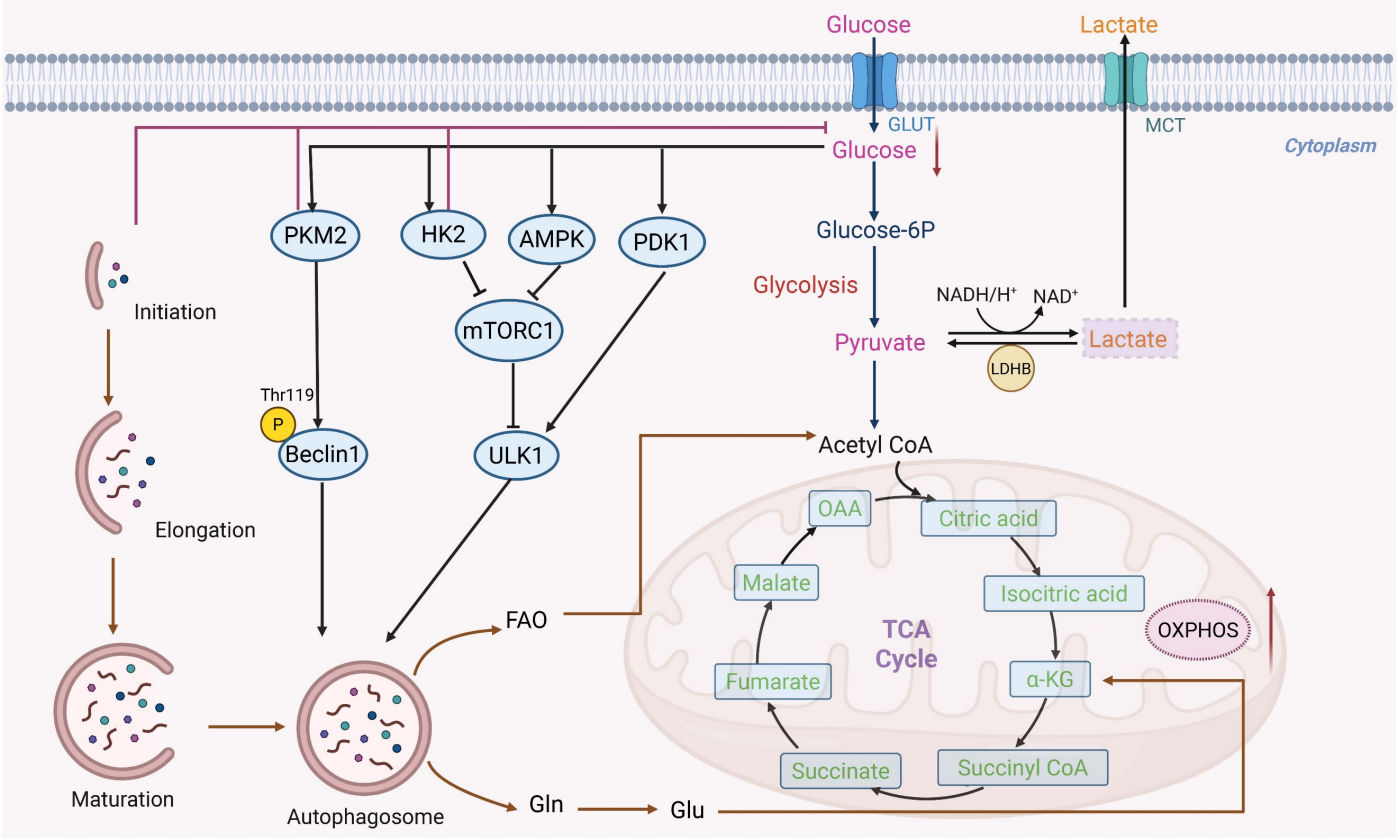
** Interplay between autophagy and glycolysis metabolism.** Enzymes linked to glycolysis, such as PKM2 and PDK1, not only control glycolysis but also trigger autophagy. While PKM2 can phosphorylate Beclin1 at the Thr119 locus, which causes the dissociation of Bcl-2 and Beclin1, activating Beclin1 and thus initiating autophagy, HK-2 binds to and inhibits mTORC1. Autophagy breaks down intracellular lipids and recycles extra or damaged organelles. When tumor cells are starved, the breakdown products that occur, such as free fatty acids and amino acids like glutamine, might give them a vital energy source.

Glycolysis catabolizes glucose into pyruvate through an intermediate step that generates ATP and nicotinamide adenine dinucleotide (NADH) 72. Compared to normal cells, tumor cells rely more on glycolysis; even in aerobic environments, cancer cells preferentially use glycolysis rather than the oxidative phosphorylation pathway to produce lactic acid and ATP, entering a metabolic state known as the Warburg effect (or aerobic glycolysis) 73. There are various benefits for cancer cells from this preferential use of glycolysis. First of all, glycolysis increases anabolic metabolism, which is essential for the rapid proliferation of cancer cells and the production of building blocks for biological macromolecules, including proteins, lipids, and nucleic acids 74. Alternatively, glycolysis produces an acidic environment that is harmful to healthy cells but has no effect on tumor cells, giving cancer cells an advantage over healthy cells in terms of growth 75. In addition, because glycolysis generates significantly fewer ROS than oxidative phosphorylation (OXPHOS), it can shield tumor cells from oxidative stress and lead to resistance to apoptosis 76. Cancer cells depend on aerobic glycolysis to generate essential biosynthetic intermediates. This metabolic dependency consequently heightens their susceptibility to glucose deprivation, which induces autophagy through the coordinated inactivation of mammalian target of rapamycin (mTOR) and activation of AMPK 77. Specifically, glucose shortage promotes the binding of HK2, an enzyme responsible for the first step of glycolysis, to mammalian target of rapamycin complex 1 (mTORC1), leading to mTOR inactivation 78. Moreover, glucose deficiency increases the AMP/ATP ratio and activates AMPK, thereby triggering autophagy 79 (Figure 3). Tumor-driving gene alterations in cancer significantly alter this glycolysis-autophagy relationship. Oncogenic signaling in KRAS-mutated pancreatic ductal adenocarcinoma not only increases glycolytic flux through HIF-1α-mediated transcriptional activation, but it also creates a novel autophagy dependency in which autophagy maintains nucleotide pools and mitochondrial function when nutrients are scarce 80. This synthetic lethal interaction creates a therapeutic vulnerability, which has been clinically developed by combining autophagy inhibitors with MEK inhibitors. In cancers driven by mutant BRAF (such as melanoma), autophagy appears to be unnecessary in baseline metabolism but becomes crucial after BRAF inhibitor treatment 81. In this case, therapy-induced senescence is associated with an increase in autophagy flux, which promotes cell survival and, eventually, resistance. These findings emphasize that the functional role of autophagy in glycolysis is dynamically determined by the carcinogenic environment and therapeutic stress. Moreover, the remodeling of the glycolysis-autophagy axis is significantly impacted by hypoxia and acidic stress in the tumor microenvironment 82. By upregulating BNIP3 and BNIP3L via HIF-1α, hypoxia stimulates mitochondrial autophagy while also increasing the development of glycolytic enzymes to maintain energy generation in low-oxygen environments 82. This coordinated response not only enables tumor cells to adapt to hypoxia but also enhances survival by limiting ROS accumulation through the clearance of damaged mitochondria 11.

More complex regulatory nodes that connect glycolysis to autophagic control have been identified in recent studies. According to a study on pancreatic ductal adenocarcinoma (PDAC), the deubiquitinase OTUD4 and the sodium-glucose transporter SLC5A2 interact to produce a stabilizing complex that preserves glycolytic flow 83. Through glycolysis-dependent pathways, this connection promotes the growth, migration, and activation of autophagy in pancreatic cancer cells. An isoform of pyruvate kinase called pyruvate kinase M2 (PKM2) functions as a slower metabolizing enzyme that turns phosphoenolpyruvate into pyruvate, building up glycolytic intermediates and promoting tumor growth 84. Beclin1 can be phosphorylated at Thr119 by PKM2 85. Beclin1 and B-cell lymphoma-2 (Bcl-2) dissociate as a result of this phosphorylation, activating Beclin1 and starting autophagy 86. Beyond this direct phosphorylation event, there is growing evidence that PKM2-dependent autophagic control is dynamically rewired under metabolic stress circumstances. In endometrial cancer, elevated glucose levels trigger the development of estrogen-related receptor α (ERRα), which activates the enzymes HK2 and hydroxymethylglutaryl-CoA synthase 1 (HMGCS1), which prevent the synthesis of cholesterol and glycolysis 87, 88. Following their binding to p62 at particular protein residues (ARG 769 on HK2 and ARG 313 on HMGCS1), these enzymes create long-lasting protein complexes that obstruct the autophagy-lysosomal pathway 87. By linking glucose sensing, lipid synthesis, and autophagic flux, ERRα-mediated metabolic reprogramming demonstrates how oncogenic transcription factors regulate multi-compartmental metabolic flexibility. Similarly, it has been demonstrated that another important glycolytic enzyme, pyruvate dehydrogenase kinase-1 (PDK1), interacts with the essential autophagy protein ULK1 to start autophagy in cancer cells 89, 90. Autophagy catabolizes damaged or unnecessary organelles and intracellular lipid droplets (LDs) when tumor cells are starving, resulting in breakdown products such as glutamine and free fatty acids, which can be essential sources of energy.

Cancer cells frequently exhibit increased aerobic glycolysis as a result of metabolic reprogramming, which leads to excessive lactate generation and export via monocarboxylate transporters (MCTs). This causes extracellular acidification and immunological suppression in the tumor microenvironment 91. But lactate is becoming more widely acknowledged as a crucial metabolic substrate and signaling molecule in cancers rather than just a waste product of metabolism. Under metabolic symbiosis, oxidative tumor cells can reimport lactate, which lactate dehydrogenase B (LDHB) then transforms into pyruvate to power the tricarboxylic acid (TCA) cycle 92. Crucially, new research indicates that autophagy may potentially be regulated by this lactate-driven metabolic circuit. For instance, it has been demonstrated that LDHB increases lysosomal acidity and autophagosome maturation to support basal autophagic flux 93. The intricate and context-dependent link between glycolysis and autophagic control in cancer metabolism is highlighted by the ongoing dispute over whether lactate-induced autophagy largely promotes tumor growth or instead constitutes a metabolic vulnerability 93. On the other hand, lactate can also be shuttled to adjacent oxidizing tumor cells, thereby facilitating metabolic symbiosis 94. Contrary to the original Warburg theory, a reduction in mitochondrial OXPHOS activity does not invariably lead to an elevation in aerobic glycolysis 95. The precise reason why rapidly proliferating cancer cells preferentially convert glucose into lactate remains an open question. One proposed explanation is that when the rate of glycolysis exceeds the capacity of mitochondrial NADH shuttles, cells sustain aerobic glycolysis, which results in NADH accumulation and subsequent lactate production 96. This reprogrammed metabolic state generates a pronounced dependence on glucose availability. Imaging techniques such as ^18^F-FDG-PET exploit this feature by detecting tumors based on their intense uptake of this glucose analog 97. Moreover, aberrant mRNA expression serves as a critical driver of aerobic glycolysis. It establishes transcriptomic signatures that are conserved across various cancer types, and these signatures offer promising opportunities for therapeutic intervention 98.

Conflicting evidence indicates that excessive autophagic activity may decrease glycolytic flux by destroying important metabolic enzymes, despite the fact that autophagy is widely thought to assist glycolytic adaptation under metabolic stress. This disparity emphasizes the need for more research on autophagy's context-dependent effects on glucose metabolism. Lipid metabolism is essential for maintaining tumor development under nutrient constraint, even if glycolysis offers quick energy and biosynthetic precursors. We'll look at how autophagy and fatty acid metabolism work together to promote the development of cancer in the next section.

### 3.2 Interplay between autophagy and lipid metabolism

Lipid metabolism, particularly the synthesis of fatty acids, plays an essential role in cellular physiology. This process converts nutrients into various metabolic intermediates, which are subsequently utilized for membrane biosynthesis, energy storage, and the production of signaling molecules 99. In cancer cells, lipid metabolism frequently undergoes reprogramming. Such reprogramming is characterized by increased uptake of fatty acids, enhanced *de novo* lipogenesis, and elevated fatty acid oxidation (FAO) 100. Recent evidence indicates that autophagy contributes to this metabolic adaptability through the selective degradation of lipid droplets, a process referred to as lipophagy. By mobilizing stored lipid reserves, lipophagy supplies free fatty acids that can be directed toward β-oxidation and ATP generation under conditions of metabolic stress 101. Although lipophagy has been widely implicated in promoting lipid utilization and supporting tumor survival, its precise role in lipid metabolic reprogramming remains incompletely understood. A key unresolved question is whether lipophagy universally facilitates tumor progression or, under certain circumstances, depletes cellular lipids and creates metabolic vulnerabilities. Experimental findings suggest that moderate lipophagy helps sustain tumor bioenergetics, whereas excessive lipid degradation may trigger lipotoxic stress and consequently impair tumor growth. Taken together, these observations indicate that the interplay between autophagy and lipid metabolism constitutes a finely balanced regulatory axis. The functional outcome of this axis is highly dependent on tumor type and the specific metabolic context.

The full oxidation of a mole of fatty acids produces around 2.5 times the ATP produced from glucose because fatty acids are a major source of energy for cancer cells. Interestingly, even in nutrient-sufficient environments, some cancer cells preferentially upregulate FAO and express large quantities of its catalytic enzymes to promote proliferation 102. Recent research discovered that palmitate, the most abundant saturated fatty acid, induces autophagy, liberates monounsaturated fatty acids, and increases agouti-related peptide (Agrp) expression in hypothalamic cells 103. Similarly, oleate, the most common monounsaturated fatty acid, promotes autophagy by raising ROS levels 104. Beyond metabolites, many enzymes involved in lipid metabolic pathways also impact autophagy. For example, some tumor cells have higher quantities of fatty acid synthase, an enzyme that produces saturated fatty acids *de novo*. This suppresses autophagy and raises p62 levels 105. A recent study discovered an atypical carnitine palmitoyltransferase 1 (CPT1) isoform, designated CPT1C, and identified it as a potential oncogene 106. It was further demonstrated that CPT1C expression promotes FAO and ATP synthesis in cancer cells, stimulates tumor development, and imparts resistance to mTORC1 inhibitors 100. Cancer reprogramming through lipid metabolism-autophagy interactions is particularly evident in the spatial heterogeneity of the tumor microenvironment 107. In hypoxic regions, HIF-1α not only induces the expression of the lipid droplet-enveloping protein PLIN2 to promote lipid storage but also upregulates BNIP3 to initiate mitochondrial autophagy 108. This allows tumor cells to retain lipids during hypoxia and then generate energy via fatty acid oxidation upon reoxygenation. This phenomenon illustrates how autophagy contributes to metabolic plasticity within the tumor microenvironment. Additionally, tumor-associated fibroblasts promote oxidative phosphorylation by releasing free fatty acids through autophagy-dependent lipolysis. These fatty acids are then transferred to nearby cancer cells via cluster of differentiation 36 (CD36). A phenomenon known as the "reverse Warburg effect" has been confirmed in a number of tumor types 109. These results imply that both tumor-intrinsic and microenvironmental effects may need to be taken into account when developing treatment approaches that target autophagy. This metabolic symbiosis creates a non-cell-autonomous therapeutic target by disrupting the tumor's energy supply by blocking CAF autophagy or fatty acid uptake by cancer cells. Furthermore, recent studies have demonstrated aberrant regulation of the molecular mechanisms that trigger lipophagy in cancer. Hypoxia-induced lipid droplet-associated protein HILPDA attracts E3 ubiquitin ligase RNF213 to the surface of lipid droplets, facilitating K63-linked ubiquitination of these droplets 110. Such ubiquitination enables the autophagy receptors p62 and NDP52 to recognize and degrade the droplets more efficiently 110. Through this ubiquitin-dependent targeting mechanism, lipid droplets are selectively degraded under metabolic stress. Furthermore, the endoplasmic reticulum-resident protein vacuolar membrane protein 1 (VMP1) coordinates autophagosome membrane formation at ER-lipid droplet contact sites, thereby physically linking lipolysis to autophagosome biogenesis 111. Disruption of these contact sites impairs both lipid droplet turnover and autophagic flux, underscoring the spatial organization of lipophagy as a critical regulatory layer.

Starvation-induced autophagy also requires stearoyl-CoA desaturase 1 (SCD1), which converts saturated fatty acids into monounsaturated fatty acids 112. Cancer cells alter their lipid uptake and utilization, relying on both endogenous and exogenous lipid sources to meet metabolic demands. Accumulating evidence indicates that lipid droplets (LDs) activate a selective autophagic pathway termed lipophagy, making it a promising therapeutic target 113. Recent mechanistic insights have revealed a bidirectional relationship: LDs not only serve as substrates for lipophagic degradation but also actively contribute to autophagosome formation by supplying membrane lipids and associated proteins 114. Specific Rab GTPases and adaptor proteins, such as Spartin, facilitate the recognition and engulfment of LDs by autophagic membranes 115. Cancer cells obtain free fatty acids (FFAs) through *de novo* synthesis or exogenous uptake. These FFAs are rapidly converted into triglycerides, which form the core of LDs. After being enclosed by autophagosomes, LDs are delivered to lysosomes, where lysosomal acid lipases hydrolyze the stored triglycerides into FFAs. These FFAs are subsequently metabolized to fuel mitochondrial OXPHOS 116. Certain FFAs, such as palmitic acid and oleic acid, can promote autophagy by inhibiting mTORC1 or via the protein kinase R (PKR)-c-Jun N-terminal kinase (JNK) pathway 117, 118. However, excessive lipid concentrations may impede autophagy by blocking autophagosome-lysosome fusion or by impairing lysosomal acidification and hydrolase activity 119. Adipose triglyceride lipase (ATGL) is a central player at the interface between lipolysis and lipophagy. As the key rate-limiting enzyme in triglyceride catabolism, ATGL hydrolyzes triglycerides into FFAs, thereby linking lipid droplet breakdown to autophagic recycling 120. Moreover, ATGL initiates lipophagy by interacting with the autophagy marker LC3, which enhances lipid breakdown 121. In direct contrast to the lipophagic pathway, fatty acid synthase (FASN) directs metabolic flux toward lipid synthesis and inhibits autophagy. FASN is the principal enzyme responsible for fatty acid production; it catalyzes the sequential condensation of acetyl-CoA and malonyl-CoA 122. Mechanistically, FASN can suppress autophagy by activating the mTOR pathway 113.

Notably, in the context of cancer treatment resistance, a unique mechanism specific to endothelial cells has recently been discovered. Tumor endothelial cells (TECs) in colorectal liver metastases upregulate the F3 protein, which blocks the autophagy-lysosomal pathway through the MAPK/JNK-MAPK/ERK-TP53 signaling axis 123. This inhibition increases FAO and fosters resistance to anti-vascular endothelial growth factor (VEGFA) therapy by preventing CPT1A protein degradation 124. Additionally, F3 encourages phagocytosis and lipid uptake, resulting in a lipid-rich milieu that permits long-term TEC growth under treatment stress 123. These findings indicate that endothelial lipid phagocytosis and fatty acid oxidation represent targetable vulnerabilities for overcoming resistance to anti-angiogenic therapies.

**Figure 4:**
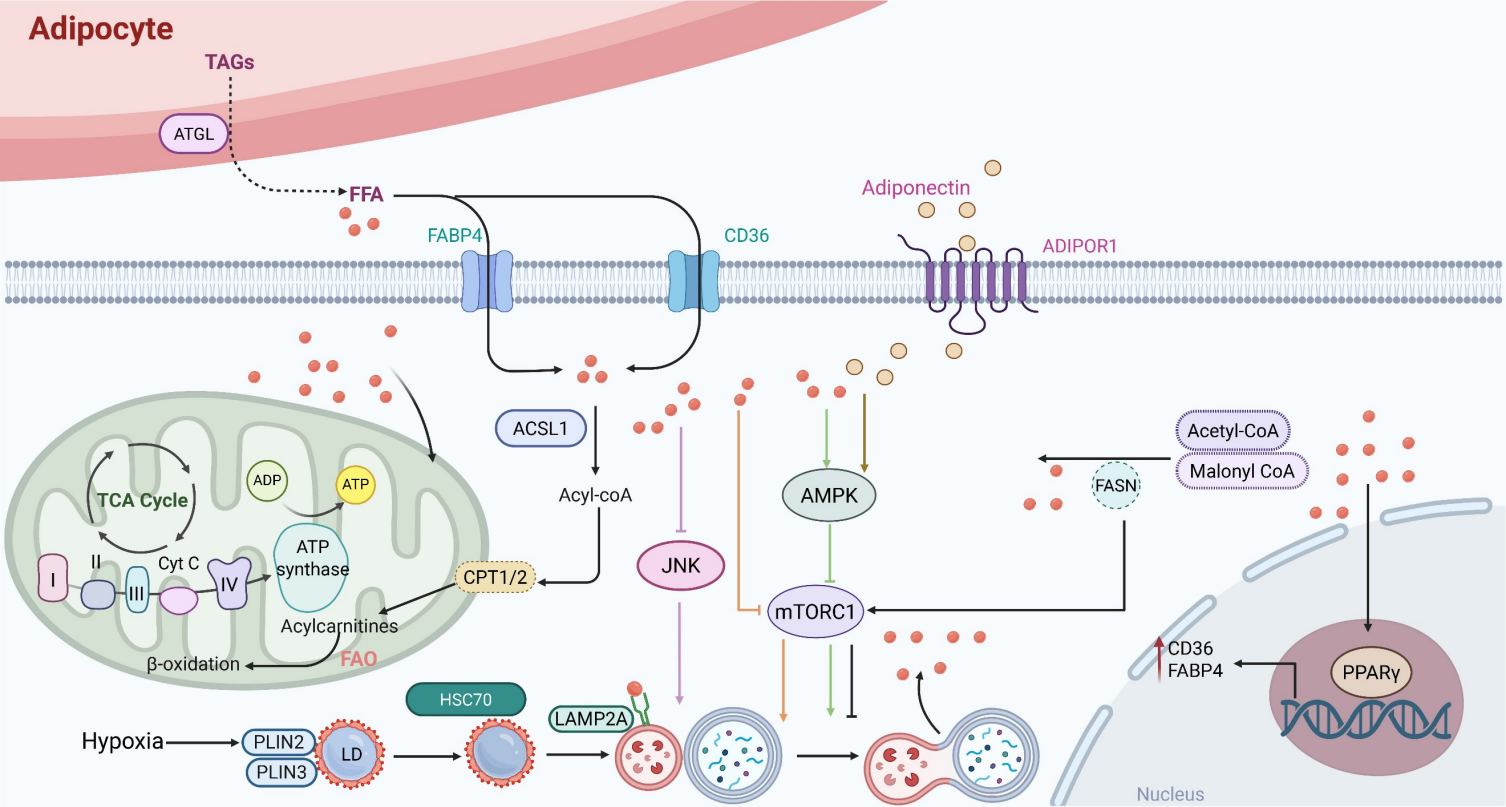
** Interplay between autophagy and fatty acid Metabolism.** FABP4 and CD36 are examples of transporter proteins that move FFAs from adipocytes to cancer cells. FAO breaks down fatty acids in cancer cells' mitochondria to provide energy. Furthermore, the cellular transcriptional network involving PPARγ and its downstream target genes FABP4 and CD36 can be activated by fatty acids, further promoting FAO and enhancing cancer cell survival. Adipocytes produce lipocalin, which interacts with its receptor ADIPOR1 to activate the AMPK pathway and ultimately cause autophagy. By stimulating the JNK pathway or blocking the mTOR pathway, FFAs also cause autophagy.

Extensive evidence indicates a comprehensive metabolic reprogramming of lipid-related processes, including synthesis, storage, and breakdown. These alterations fuel tumor progression and sustain oncogenesis 125. Fatty acids produced by adipocytes promote FAO and cancer cell survival by activating peroxisome proliferator-activated receptor γ2 (PPARγ) and the transcription of its downstream target genes, such as fatty acid-binding protein 4 (FABP4) and CD36, in cancer cells 113. For instance, transporter proteins such as FABP4 and CD36 carry fatty acids into acute myeloid leukemia (AML) cells 126. Notably, adiponectin secreted by adipocytes binds to its receptor, adiponectin receptor 1 (ADIPOR1), thereby activating the AMPK pathway and inducing autophagy 127. It has been demonstrated that adiponectin inhibits glycolysis and activates the AMPK/ULK1 signaling pathway, consequently suppressing the proliferation of PCa cells 69. The degradation of perilipins (PLINs), which are core scaffold proteins on the surface of lipid droplets, represents a key prerequisite that licenses lipolysis 128. PLINs are degraded by CMA. This process is triggered by the binding of heat shock cognate protein 70 (HSC70) to specific pentapeptide motifs on PLIN2 and PLIN3. Subsequently, the HSC70-PLIN complex binds to lysosome-associated membrane protein 2A (LAMP2A) and is taken up into lysosomes, where it is degraded 128, 129 (Figure 4). In this context, a key adaptive change is the activation of lipogenic pathways. Through these pathways, cancer cells boost the production of fatty acids and other lipid molecules to support their rapid proliferation. Increased lipogenesis is frequently linked to the activation of essential enzymes, such as ATP citrate lyase (ACLY) and acetyl-CoA carboxylase (ACC), under the influence of carcinogenic signaling pathways, including phosphatidylinositol 3-kinase (PI3K), protein kinase B (Akt), mTOR, and Myc 130. Hypoxia is regarded as a defining feature of cancer and plays a significant part in the metabolic reprogramming of tumor cells 131. It promotes the accumulation of triglycerides and LDs by upregulating Lipin1, a key enzyme catalyzing the conversion of phosphatidic acid. Furthermore, hypoxia enhances lipid storage within these droplets through the induction of the lipid droplet coating protein PLIN2 132. The survival of cells exposed to hypoxia-reoxygenation *in vitro* is decreased when lipid storage is inhibited, which can significantly hinder cancer *in vivo*. Therefore, exploring the changes in lipid metabolism in cancer cells is of great significance for the development of new cancer treatment methods.

### 3.3 Interplay between autophagy and amino acids metabolism

In addition to reprogramming their metabolism of fats and carbohydrates, tumor cells have a noticeably higher need for amino acids in order to sustain their fast growth and division 133. Amino acid metabolism, including that of glutamine, leucine, and proline, is closely linked to the development and survival of cancer cells 134. As the most prevalent amino acid in human blood, glutamine is essential for maintaining the regular operations of many cell types 135. Nucleotide synthesis is the main function of glutamine metabolism, while glucose provides carbon and glutamine supplies nitrogen 136. Glutaminase (GLS) is the enzyme that normal cells use to make glutamine. Nevertheless, the incapacity of tumor cells to generate enough glutamine to meet their high growth requirements leads to a glutamine-dependent phenomenon 136. The glutamine transporter proteins sodium-coupled neutral amino acid transporter 2 (SNAT2) and solute carrier family 1, member 5 (SLC1A5) are increased as a result of hypoxia, preventing pyruvate from entering the TCA cycle 137. Glutamine metabolism plays a key role in energy production, antioxidant defense and cancer cell growth. After GLS converts glutamine to glutamate, either glutamate dehydrogenase (GLUD) or aminotransferase produces α-ketoglutarate, which enters the TCA cycle to supply cellular energy needs 70. In addition, glutamine is an important raw material for the production of glutathione (GSH) and is involved in stabilizing ROS 138. GLS1, often upregulated by c-Myc in cancer cells, promotes GSH synthesis to reduce ROS and protect cells from oxidative damage 139. Collectively, glutamine metabolism provides the raw material for hyperactive glycolysis and OXPHOS in cancer cells. It can also cause tumor cells to become resistant to chemotherapy drugs by disrupting the balance of sugar, lipid and protein metabolism 140. Proliferative cancer cells can compete with normal cells for circulating glutamine, and there are significant differences in glutamine metabolism between organs at various stages of tumor progression, suggesting that targeted regulation of glutamine metabolism is a promising approach to tumor treatment.

**Figure 5:**
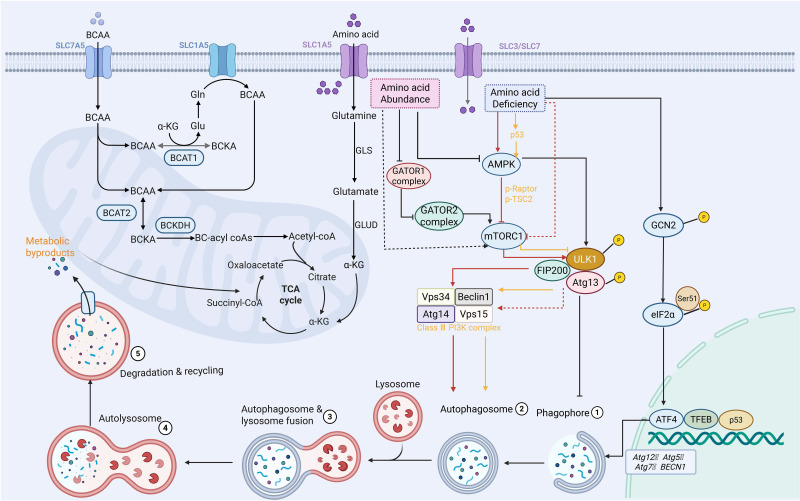
** Interplay between autophagy and amino acid metabolism.** The GCN2/eIF2α/ATF4 pathway, AMPK, and the mTORC1 pathway are the primary regulators of amino acid homeostasis. By regulating protein synthesis and autophagy, these signaling pathways collaborate to monitor intracellular amino acid levels and maintain homeostasis *in vivo*.

Leucine, isoleucine, and valine make up branched-chain amino acids (BCAAs), which are the most prevalent necessary amino acids. They play a pivotal role in regulating energy homeostasis, nutritional metabolism, immunity, and various diseases in both humans and animals 141. BCAAs are not precursors for biosynthetic nitrogen compounds but function as signaling molecules regulating gluconeogenesis, lipid synthesis, and protein anabolism. Consequently, BCAAs are also indispensable for cancer cells, which thus enhance their uptake and utilization to support proliferation 142. The levels of BCAAs are primarily regulated by the cytoplasmic branched-chain amino acids transaminase isoenzyme 1 (BCAT1), the mitochondrial branched-chain amino acids transaminase isoenzyme 2 (BCAT2), and the branched-chain α-ketoacid dehydrogenase complex (BCKDH) 143. Nitrogen is transferred to α-ketoglutarate (αKG) by BCAT1 and BCAT2, resulting in glutamate and the specific branched-chain ketone acid (BCKA). After that, the BCKDH breaks down these ketone acids to create branched-chain acyl-CoA (BC-CoA). For energy production and macromolecular synthesis, this CoA can be further metabolized in several stages to produce acetyl-CoA and succinyl-CoA, TCA cycle intermediates 144 (Figure 5). Recent studies have highlighted cancer type-specific roles of branched-chain amino acid (BCAA) metabolism in autophagy regulation. In PCa, BCAT2 is markedly upregulated and correlates with tumor progression and poor prognosis. Mechanistically, BCAT2 interacts with PCBP1 at the Leu239 site to activate PI3K/AKT signaling, thereby suppressing autophagy-associated apoptosis and ferroptosis in PCa cells 143. Consequently, BCAT2 is identified as a potential diagnostic and prognostic biomarker as well as a promising therapeutic target in PCa. In HCC, BCAAs suppress autophagy through mTORC1-dependent phosphorylation of ULK1 at Ser757, which impairs autophagosome formation and stabilizes the tumor suppressor PDCD4 by preventing its autophagic degradation. This mechanism suggests that BCAAs may exert antitumor effects by coordinately modulating autophagy and tumor suppressor pathways 145.

Autophagy is traditionally regulated by amino acids. The transcription of autophagy genes, such as Atg12, Atg5, Atg7, and Beclin1, can be triggered by the general control non-derepressible 2 (GCN2), eukaryotic initiation factor 2α (eIF2α), and activating transcription factor 4 (ATF4) pathways when amino acid deficiency occurs. This process elevates cytoplasmic amino acid levels and triggers the initiation of autophagy 146. It should be noted that AMPK does not directly detect amino acid availability. However, when amino acids become scarce, protein synthesis and mitochondrial function are compromised. As a consequence, the AMP/ATP ratio rises, which in turn activates AMPK 147. At the same time, mTORC1 activity is directly inhibited by the lack of amino acids, particularly under conditions of leucine or arginine depletion. This inhibition facilitates the nuclear translocation of TFEB and TFE3. Once in the nucleus, these transcription factors drive increased expression of autophagy-related and lysosomal genes 89. Additionally, via phosphorylating Raptor and tuberous sclerosis 2 (TSC2), AMPK strengthens this suppression of mTORC1 148. Together, these actions ensure enhanced ULK1 activation and autophagic induction under amino acid scarcity 149. Recent advances have further elucidated the intricate molecular machinery governing amino acid sensing. The gap activity towards rags (GATOR) complex, a crucial amino acid sensor on the lysosomal membrane, is activated in nutrient-rich environments, especially when leucine and arginine are abundant 150. Specifically, the abundance of amino acids suppresses the activity of the GATOR1 complex, thereby activating GATOR2 to promote the transition of Ras-related GTP-binding proteins (Rag GTPases) into their active state on the lysosomal membrane 151. These active Rag complexes then recruit mTORC1 to the lysosomal surface, where it interacts with GTPase ras homolog enriched in brain (Rheb) to achieve full activation of mTORC1 150. In the end, active mTORC1 phosphorylates the essential elements of autophagy initiation, including ULK1, Atg13, FIP200, and Atg14, which stops phagophore production and suppresses autophagic initiation 152. Despite substantial evidence supporting the role of autophagy in sustaining amino acid pools, emerging studies suggest that excessive reliance on autophagy-derived nutrients may create metabolic dependencies that could be therapeutically exploited. However, the extent of this vulnerability remains to be systematically characterized.

Beyond the canonical mTORC1 pathway, emerging evidence indicates that amino acids can regulate autophagy through mTORC1-independent mechanisms. Recent work has shown that glutamine acts as a signaling molecule capable of binding directly to HSC70. This binding occurs independently of known glutamine metabolic or signaling routes 153. The interaction between glutamine and HSC70 inhibits the degradation of the deubiquitinating enzyme OTUD4. As a result, lactate dehydrogenase A (LDHA) is stabilized through the removal of its ubiquitin chains 154, 155. This process suppresses the microautophagy-lysosomal pathway, enhances lactate synthesis, and reduces the expression of interferon-β and its targets. All of these changes are hallmarks of immunogenic cell death. The tumor suppressor p53 is a stress-responsive transcription factor. It exerts complex and context-dependent regulation over both autophagy and amino acid metabolism. Its function is critically influenced by subcellular localization 156. In the nucleus, p53 promotes autophagy by transcriptionally regulating the mTOR pathway and key autophagy-related genes. In the cytoplasm, however, it inhibits autophagy under basal conditions 157. Through the control of target genes such as glutaminase 2 (GLS2), p53 influences cellular energy supply and redox balance. It also regulates the expression of several important enzymes involved in amino acid metabolism, particularly glutamine metabolism 158. Conversely, the availability of amino acids inversely regulates the activity and stability of p53. In response to amino acid shortage or cellular stress, p53 transcriptionally up-regulates tuberous sclerosis complex 2 (TSC2), also known as phosphatase and tensin homolog (PTEN), as well as AMPK 157.

In conclusion, cancer cells are able to maintain their bioenergetic and biosynthetic needs under stress due to the complex interactions between autophagy and important metabolic pathways, such as glycolysis, lipid metabolism, and amino acid metabolism. This metabolic flexibility, supported by autophagy-mediated recycling and signaling integration, drives tumor progression and therapy resistance. Consequently, therapeutic strategies that target the autophagy-metabolism axis—a critical driver of cancer progression—have attracted considerable interest due to their potential to disrupt tumor adaptation. The following section will discuss emerging small-molecule agents that exploit this vulnerability.

### 3.4 Interplay between autophagy and other metabolic processes

**Figure 6:**
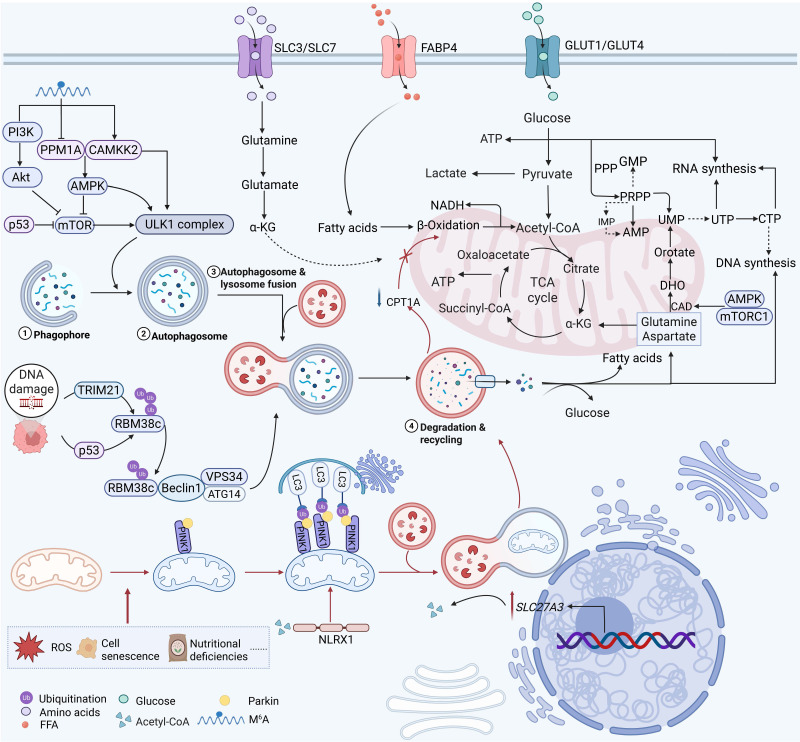
** Interactions between autophagy and nucleotide metabolism and mitochondrial metabolism.** Under stress conditions such as DNA damage, cellular senescence, and nutritional deficiency, autophagy-related molecules (e.g., p53, TRIM21) interact with metabolic pathways. Glucose, glutamine, and fatty acids enter the cell via their respective transporters and are converted into ATP, NADH, and metabolic intermediates through glycolysis, the citric acid cycle, and β-oxidation, collectively regulating nucleotide synthesis. Mitochondrial autophagy maintains a healthy mitochondrial network and enables metabolic flexibility under stress conditions. CAD, carbamoyl phosphate synthetase II; DHO, dihydroorotate.

Beyond the classical metabolic pathways discussed above, accumulating evidence points to nucleotide metabolism as another critical component of the autophagy-metabolism network. Cancer cells commonly exhibit excessive synthesis and utilization of nucleotide triphosphates (NTPs) and their deoxyribonucleotide counterparts (dNTPs) 159. The synthesis of purine and pyrimidine nucleotides proceeds through two distinct routes. One is the *de novo* pathway, which incorporates small precursors into nucleotides via a series of energy-intensive, multi-step enzymatic reactions. The other is the nucleoside/nucleobase salvage pathway, where nucleosides or nucleobases are converted into the corresponding nucleoside monophosphates (NMPs) through a single phosphorylation or phosphoribosyltransferase reaction 160. For both pyrimidines and purines, *de novo* synthesis involves multiple complex steps. These steps convert amino acids and phosphoribosyl pyrophosphate (PRPP) into uridine monophosphate (UMP) or inosine monophosphate (IMP), both of which participate in nucleic acid metabolism 160. The dynamic crosstalk between autophagy and nucleotide metabolism has emerged as an important determinant of cancer cell adaptation and therapeutic resistance 161. Mechanistically, a metabolic change is brought about by persistent autophagy suppression, such as in pancreatic ductal adenocarcinoma cells that have become resistant to hydroxychloroquine or ULK1/2 inhibitors. This shift moves the cells from *de novo* pyrimidine synthesis toward preferential utilization of the salvage pathway. It is characterized by elevated aspartate levels and altered pyruvate metabolism 162. Such metabolic rewiring creates a vulnerability to pyrimidine analogs like gemcitabine and trifluridine, highlighting a compensatory relationship between autophagic flux and the maintenance of nucleotide pools. At the same time, autophagic regulation and nucleotide metabolism are connected via RNA epigenetics, specifically N6-methyladenosine (m^6^A) modification 163. The research that is now available indicates that the m6A modification can influence the start and length of autophagy by controlling the expression of FIP200, ULK1, and ATG5/ATG7. Furthermore, it has been observed that m6A modification regulates the AMPK/AKT pathway, which is crucial for regulating autophagy 164. Specifically, a reduction in m^6^A modification suppresses the expression of PPM1A (an AMPK inhibitor) while promoting the expression of CAMKK2 (an AMPK activator). These changes lead to the activation of autophagy 165. Reduced m^6^A modification levels may activate the AKT signaling pathway 166. In response to DNA damage, the p53-inducible isoform RBM38c undergoes K63-linked polyubiquitination catalyzed by TRIM21, enhancing its interaction with Beclin1 and promoting assembly of the ATG14-containing VPS34 complex to initiate protective autophagy (Figure 6). This ubiquitin-dependent mechanism couples genotoxic stress recognition to autophagosome nucleation, and its disruption sensitizes cancer cells to DNA-damaging agents 167. In a study on triple-negative breast cancer (TNBC), curcumin's reactive oxygen species interact with LC3 to cause the inner nuclear membrane protein Lamin B1 to degrade, discharging damaged DNA into the micronucleus and initiating the cGAS-STING immunological pathway. This mechanism connects immune activation, autophagy clearance, and nucleotide damage monitoring 168. Furthermore, nicotinamide mononucleotide (NMN), the precursor of NAD⁺, directly activates the AMPK/mTOR axis to promote autophagy while concurrently modifying redox balance and ferroptosis sensitivity, demonstrating how a crucial nucleotide cofactor combines energy sensing with autophagic flux 169.

By selectively removing malfunctioning organelles through mitophagy, autophagy not only provides nucleotide precursors through nucleic acid catabolism but also acts as a key regulator of mitochondrial homeostasis, maintaining cellular energy production and redox balance under metabolic stress conditions. In order to promote the growth and survival of cancer cells, mitochondria work as key metabolic hubs where oxidative phosphorylation, TCA cycle activity, nucleotide biosynthesis, and redox homeostasis come together 170. By eliminating dysfunctional mitochondria that generate excessive ROS and fail to meet bioenergetic demands, mitophagy preserves a healthy mitochondrial network and enables metabolic flexibility under stress conditions such as nutrient deprivation, hypoxia, and therapeutic pressure 11. In cancer, autophagy and mitochondrial metabolism interact in both directions and are dynamically controlled 171, 172. On the one hand, autophagic activity is directly impacted by mitochondrial metabolic states. Cytosolic acetyl-CoA is a signaling molecule that directly regulates mitophagy by binding to the NOD-like receptor (NLR) family member X1 (NLRX1), according to a ground-breaking discovery 173. Surprisingly, this acetyl-CoA-NLRX1 axis functions independently of the traditional AMPK and mTOR signaling pathways, exposing a direct "metabolite-sensing" mechanism that links mitochondrial quality management to cellular energy status. On the other hand, to promote cancer growth and treatment resistance, mitophagy actively modifies mitochondrial metabolic networks. Upregulation of SLC27A3, a lipid metabolism gene linked to a poor prognosis in clear cell renal cell carcinoma, increases the formation of acyl-CoA, which causes the dissipation of the mitochondrial membrane potential and the development of ROS that initiate PINK1/Parkin-mediated mitophagy 174. By consuming CPT1A in mitochondria and driving acyl-CoA toward lipid droplet synthesis and storage, this mitophagic response simultaneously stops fatty acid oxidation and modifies the lipid metabolic landscape to promote tumor growth and provide resistance to the tyrosine kinase inhibitor pazopanib 174. Targeting the autophagy-mitochondria metabolic axis has been shown to have therapeutic promise. Icaritin, a naturally occurring substance, causes ROS generation and mitochondrial damage in HCC, which sets off PINK1/Parkin-mediated mitophagy 175.

Interestingly, icaritin-induced cell death and anticancer efficacy are greatly increased when autophagy or mitophagy is inhibited, suggesting that mitophagy functions as a cytoprotective mechanism that restricts therapeutic response 175. Similarly, via controlling energy metabolism, oxidative stress, and cell-fate decisions, mitophagy in breast cancer plays simultaneous roles in tumor formation, metastasis, and treatment resistance 172. These results lend credence to the idea that mitophagy inhibitors should be used in conjunction with traditional treatments to combat medication resistance.

Overall, a highly coordinated adaptive network that allows cancer cells to withstand metabolic stress, avoid apoptosis, and withstand treatment is shown by the interplay between autophagy and nucleotide and mitochondrial metabolism. In order to sustain growth and energy production, autophagy, on the one hand, encourages nucleotide recycling and mitochondrial quality control. However, these organelles generate metabolic signals that reciprocally control autophagy activity, creating a potent homeostatic loop. There are important therapeutic ramifications to this bidirectional regulation. Drug resistance may be overcome by controlling mitochondrial autophagy, either by blocking protective mitochondrial autophagy or by utilizing metabolite-sensing vulnerability (such as the acetyl-CoA-NLRX1 route).

Despite significant progress in our understanding of the relationship between autophagy and metabolism, many important questions still remain unanswered. Firstly, the context-dependent dual function of autophagy, tumor-promoting versus tumor-suppressive, presents a significant challenge for therapeutic intervention, as clinical outcomes depend on the timing and duration of autophagy regulation 176. Moreover, the precise molecular processes by which metabolic stress signals are integrated to activate selective autophagic responses are not fully understood, with accumulating evidence indicating cell-type and stress-specific differences 177. Following that, the metabolic requirements regulated by autophagy vary significantly between cancer types and even within the same tumor, hindering the development of generally applicable treatment techniques 178. Importantly, whether autophagy primarily supports tumor metabolic fitness or instead creates metabolic liabilities under specific conditions remains highly context-dependent. Furthermore, new research indicates that different types of autophagy may have different, sometimes conflicting, metabolic impacts, underscoring the need for a more complex and comprehensive framework. These unresolved problems underscore the need for a deeper understanding of the autophagy-metabolism axis, which is what this study attempts to critically examine. Collectively, these emerging metabolic dimensions further support the concept that autophagy functions as a central integrator of metabolic networks rather than a pathway acting on isolated metabolic processes. Nevertheless, it is still unclear how these pathways are arranged mechanistically and how much each contributes to the development of tumors.

## 4. Small-molecule drugs linking autophagy to metabolism in cancers

**Figure 7:**
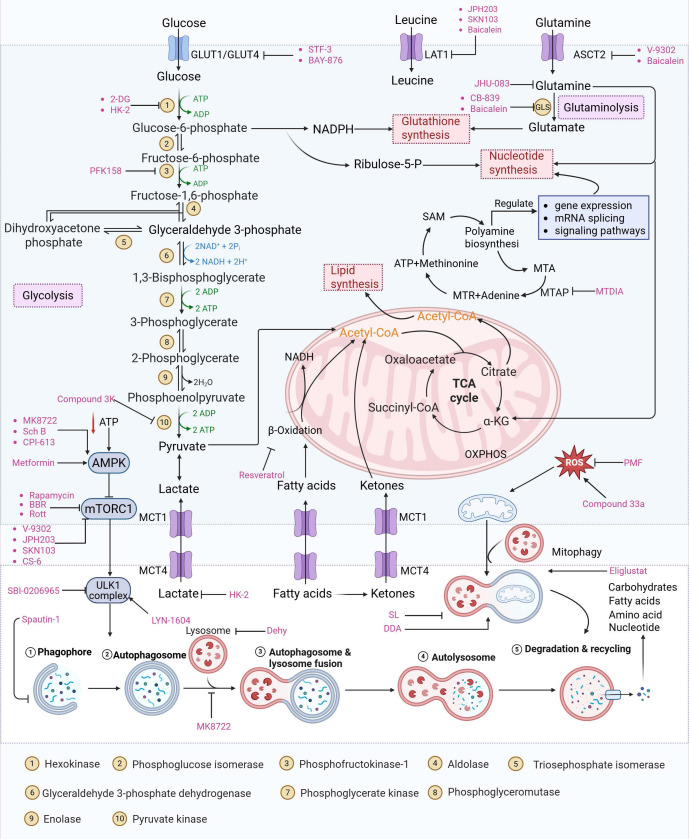
** Overview of small molecule drugs targeting the autophagy-metabolism axis and their mechanism of action.** This figure comprehensively summarizes the current small molecule drugs targeting the interaction network of autophagy and metabolism in cancer, covering three major pathways: glycolysis, fatty acid metabolism, and amino acid metabolism, as well as other metabolic pathways such as nucleotide metabolism and mitochondrial metabolism. It shows the key targets of the drugs and their regulatory effects on autophagy (activation or inhibition), providing a theoretical basis for combined treatment strategies.

An intriguing treatment avenue is the complex interaction between autophagy and cancer metabolism. Autophagy provides metabolic precursors, including lipids and amino acids, to maintain biosynthetic pathways under food stress, and metabolic reprogramming reciprocally adjusts autophagic flow 37, 179. The autophagy-metabolism axis is a viable target for anticancer therapies because of its bidirectional communication. A crucial part of this control is played by nutritional sensors: while nutrients are plentiful, mTORC1 inhibits autophagy, but under stress, it becomes inactive, enabling ULK1-driven autophagosome formation and metabolic recycling 180. In the meantime, when energy is depleted, AMPK is activated, phosphorylating ULK1 to start autophagy and simultaneously repressing mTORC1 to move cells toward catabolism 179. Furthermore, selective autophagic processes such as mitophagy preserve mitochondrial integrity, supporting oxidative phosphorylation and mitigating reactive oxygen species in malignancies, including PDAC 102, 181. Pharmacologically targeting autophagy thus offers a rational approach to disrupt metabolic adaptability in tumors, such as glutamine catabolism and mitochondrial metabolism, while potentially overcoming conventional therapy resistance and minimizing off-target effects 182-184. The mechanisms, effectiveness, and possibilities for combination therapy of small-molecule medicines that target both autophagy and important metabolic pathways are extensively reviewed in this section (Figure 7).

### 4.1 Small-molecule drugs targeting the glycolytic pathway and autophagy

As a central metabolic pathway, glycolysis is frequently dysregulated in cancers, fueling tumorigenesis, proliferation, and therapy resistance 185, 186. The bioenergetic and metabolic basis of malignancies is disrupted by targeting this pathway, a vulnerability highlighted by recurring mutations in important enzymes such as succinate dehydrogenase (SDH), fumarate hydratase (FH), and isocitrate dehydrogenase (IDH) 187-189. This section discusses small-molecule drugs that exploit this vulnerability to treat cancers by targeting glycolytic or autophagic activity.

As an HK2 inhibitor, 2-deoxy-D-glucose (2-DG) effectively prevents glycolysis by blocking HK-mediated phosphorylation, which restricts the growth of tumor cells 190. A study showed that 2-DG triggered glucose deprivation without altering other nutrients or metabolic pathways, activated AMPK, increased ROS in cancer cells, and triggered autophagy 191. To enhance the anti-tumor effect of 2-DG, researchers have proposed to combine 2-DG with autophagy inhibitors 192. For example, combining 2-DG-mediated starvation therapy with an autophagy inhibitor, black phosphorus nanosheet can disrupt the autophagy mechanism of tumor cells and maximize the therapeutic effect 193. In short‌, 2-DG exerts antitumor effects by inhibiting glycolysis and modulating pro-survival autophagy. Combining 2-DG with autophagy inhibition may therefore enhance its therapeutic efficacy 190. Another HK2 inhibitor is 3-bromopyruvic acid (3-BrPA), a pyruvate analog that suppresses cancer cell energy metabolism and induces apoptosis by activating autophagy in myeloma cells under hypoxic conditions 71. Furthermore, a study showed that 3-BrPA suppresses the growth of TNBC cells by downregulating c-Myc, which lowers HK2 expression, hinders glycolysis (including lactate formation, ATP generation, and HK activity), and encourages mitochondria-mediated apoptosis 194. Similarly, in thyroid cancer, 3-BrPA-mediated glycolysis inhibition significantly curbs tumor cell proliferation and invasion, consistent with its role in disrupting energy metabolism 195. Rapamycin, a classical mTOR inhibitor that activates autophagy by suppressing mTOR signaling, exhibits synergistic antitumor activity when combined with 3-BrPA, concurrently modulating ATGs and intracellular glucose metabolism in high-risk neuroblastoma models 196, 197.

Metformin has shown promise as an anticancer drug against a variety of cancers in addition to its traditional usage in type 2 diabetes 198, 199. It works by lowering cellular energy and triggering AMPK, which in turn inhibits mTOR and induces autophagy 200. By changing the metabolic landscape, metformin can stop the growth of insulin-sensitive cancers and increase their susceptibility to conventional therapies 201. The investigation of metformin in combination therapy has been spurred by these mechanistic findings, particularly for malignancies like breast and colon cancer that have changed metabolic pathways 202, 203, and have inspired a number of related clinical trials 204. Berberine (BBR), an isoquinoline alkaloid obtained from Berberis vulgaris, has been used extensively in traditional Chinese medicine to treat diabetes and diarrhea. It also possesses anticancer properties against a range of cancer cell types, including human glioblastoma cells 205, colorectal 206, lung 207, prostate 208, and ovarian cancers 209. BBR suppresses tumor cell invasion, an energy-demanding process, by disrupting glycolytic energy production and impairing mitochondrial function 210. In glioblastoma multiforme (GBM), BBR perturbs cellular metabolism, curbing glycolysis, altering mitochondrial dynamics, and enhancing autophagic flux, collectively reducing invasiveness and promoting cell death 211. By controlling the Akt/AMPK/mTOR pathway, compound 3K, a specific PKM2 inhibitor, impairs glycolysis and triggers autophagy, ultimately leading to the death of ovarian cancer cells 212. PKM2 catalyzes the terminal, rate-limiting step of glycolysis, conversion of phosphoenolpyruvate to pyruvate, and thereby supports aerobic glycolysis and rapid cancer cell proliferation 213. However, its therapeutic benefit in clinical settings has yet to be clarified, although current evidence suggests considerable potential for further development. Polymethoxyflavonoids (PMFs) are a kind of flavonoid that exhibit broad-spectrum anticancer and chemosensitizing activities 214, 215. According to a study, PMFs reverse chemoresistance in colorectal cancer by suppressing aerobic glycolysis, reducing ROS and autophagosome formation 216.

With glucose uptake via glucose transporter proteins, particularly glucose transporter protein 1 (GLUT1), acting as a crucial regulatory node in glycolytic flux, the Warburg effect highlights the extreme dependence of cancer cells on aerobic glycolysis 217, 218. In a dose-dependent manner, pharmacological GLUT1 inhibitors such as STF-31 219 and BAY-876 220 successfully decrease glucose uptake, resulting in intracellular glucose scarcity and energy depletion. This metabolic stress causes cell cycle arrest and protective autophagy in thyroid cancer cells by activating AMPK in response to increased AMP: ATP and ADP: ATP ratios 221. Furthermore, GLUT1 inhibitors exhibit synergistic potential with conventional chemotherapeutics 222. For instance, they enhance the antiproliferative efficacy of Lenvatinib and sensitize esophageal cancer cells to cisplatin 223. Similarly, co-treatment with BAY-876 and docetaxel augments apoptosis in lung cancer models 224. These findings demonstrate the usefulness of GLUT1 inhibitors as possible cancer combination treatment options. Beclin1 and vacuolar protein sorting 34 (VPS34) are ubiquitinated and degraded as a result of the deubiquitination activity of ubiquitin-specific peptidase 10 (USP10) and USP13, which is blocked by the strong autophagy inhibitor spautin-1 225. In addition to its conventional function, Spautin-1 inhibits mitochondrial complex I activity, which in turn lowers the unfolded protein response (UPR) and reduces the survival of cancer cells during glucose deprivation 226. ULK1 is a master regulator that transmits metabolic stress signals to initiate autophagy and maintain glucose metabolism 227, 228. Glioblastoma and lung cancer cells undergo apoptosis and autophagy inhibition when exposed to SBI-0206965, a selective small molecule inhibitor of ULK1 kinase 229. Additionally, in non-small cell lung cancer (NSCLC) cells, it has been shown to increase cisplatin sensitivity 230. Furthermore, in clear cell renal cell carcinoma (ccRCC), SBI-0206965 triggers apoptosis by dual inhibition of autophagy and the PPP—a glycolytic branch critical for NADPH production and redox balance 231, 232. (Table 1).

In summary, blocking the glycolysis-autophagy axis using inhibitors of HK2, PKM2, GLUT1, ULK1, and other proteins is a proven method of interfering with the anabolic and bioenergetic processes that are essential for tumor growth.

### 4.2 Small-molecule drugs targeting lipid metabolism and autophagy

As a lipolytic mechanism, autophagy encourages lipid turnover to support cancer stem cells (CSCs), strengthening their stemness, survival, and chemoresistance while promoting tumorigenesis and metastasis 128. Thus, targeting this process represents a promising strategy to overcome therapy resistance, as demonstrated by the autophagy inhibitor verteporfin, which sensitizes pancreatic ductal adenocarcinoma to gemcitabine 233. Targeting autophagy to modulate lipid metabolism represents a promising strategy to overcome therapy resistance in CSCs. By inhibiting the PI3K/Akt/mTOR pathway, the plant-derived substance Rottlerin (Rott) causes lipid modification of LC3 and triggers autophagy and death in prostate CSCs 234. Rott's extensive therapeutic potential extends beyond prostate cancer, since it induces mTORC1-independent autophagic cell death in apoptosis-resistant MCF-7 breast cancer cells 235. Schisandrin B (Sch B), a bioactive dibenzocyclodiene derived from Schisandra chinensis, decreases hepatic steatosis in non-alcoholic fatty liver disease (NAFLD) via inducing autophagy through the AMPK/mTOR pathway. Concurrently, Sch B upregulates key enzymes involved in fatty acid oxidation and ketone production, indicating a coordinated enhancement of lipid catabolism 236. These findings position Sch B-induced autophagy as a central mechanism responsible for remodeling hepatic lipid and amino acid metabolism. The natural sesquiterpene lactone Dehydrocostus lactone (Dehy), derived from Saussurea lappa Clarke, exhibits potent anti-gastric cancer (GC) activity by targeting lipid metabolism 237. It suppresses aberrant *de novo* fatty acid synthesis through the IKKβ/ACLY axis, leading to lipid depletion that triggers a compensatory autophagy initiation in GC cells 238. Dehy, however, simultaneously inhibits autophagic flow at the stage of lysosomal breakdown. Dehy's dual mode of action against GC is highlighted by the fact that insufficient lipid supply ultimately fails to meet the demands of rapid cancer growth and produces apoptosis 239. In human epithelial ovarian cancer (EOC), MK8722 (C_24_H_20_ClN_3_O_4_), a very effective and direct small molecule activator of AMPK in mammals, reduces malignant characteristics and causes cell death via a complex process involving both autophagy and lipid metabolism 240-243. Mechanistically, MK8722 triggers autophagosome formation via PI3K/AKT/mTOR inhibition but concurrently blocks lysosomal fusion, leading to FASN downregulation, defective lipophagy and mitophagy, and accumulation of autophagosomes, LDs, and ROS that ultimately induce lethal metabolic stress 243. Furthermore, by activating AMPK, which targets lipid metabolism, the thioctic acid analog CPI-613 (Devimistat) suppresses ACC. In pancreatic cancer cells, this dual effect inhibits *de novo* lipogenesis, promotes autophagy, and initiates ROS-dependent death 244-246. It has been demonstrated that LYN-1604, a new small-molecule agonist of ULK1, modulates lipid metabolism via ULK1-driven autophagy, improving obesity-related heart dysfunction by lowering lipid buildup 247, 248. Nevertheless, there is still little concrete proof that LYN-1604 promotes fatty acid oxidation or inhibits lipid production in lipid-addicted tumors. To clarify the molecular underpinnings of LYN-1604 in lipid metabolic reprogramming, more research is necessary.

As previously stated, LDs, which are common eukaryotic organelles made up of a phospholipid monolayer encircling a neutral lipid core, build up in a variety of malignancies. Increased activity of the glycolytic regulator PFKFB3, which regulates glycolytic flux through fructose-2,6-bisphosphate (F-2,6-BP) production, is correlated with greater LD levels in ovarian and cervical cancer models 249, 250. By decreasing LD accumulation through increased autophagic flux and lipophagy, PFK158, a PFKFB3 inhibitor, inhibits tumor development and eventually induces apoptosis 251. Furthermore, etomoxir, a derivative of epoxyalkanecarboxylic acid, prevents fatty acids from being transported to the mitochondria and from being further catabolized by β-oxidation by permanently inhibiting the activity of CPT1. This compels cancer cells to reduce metabolic load by using autophagy to break down lipid droplets 252. Unfortunately, hepatotoxicity in congestive heart failure patients led to the cessation of its clinical development 253. Unlike most normal mature cells that get their fatty acids from circulation, cancer cells enhance *de novo* lipogenesis, which is mostly regulated by the master transcription factor sterol regulatory element-binding protein 1 254, 255. This important pathway is targeted by the naturally occurring polyphenol resveratrol, which is found in many foods and plants 256, 257. Resveratrol has recently been demonstrated to restrict lipid metabolism and activate autophagy in oral cancer cells through downregulation of SREBP1, indicating a unique mechanism of its anticancer effect beyond its well-established activities in inducing death in cancer cells 258, 259. (Table 2).

Lipid metabolism reprogramming is essential for tumorigenesis, chemotherapy resistance, and the maintenance of cancer stem cell properties. Emerging research gathered in this section highlights the therapeutic potential of employing small-molecule drugs to target the connection between autophagy and lipid metabolic pathways in cancer. This complex interrelationship can be pharmacologically disrupted, which offers intriguing ways to interfere with tumor metabolic resilience.

### 4.3 Small-molecule drugs targeting amino acid metabolism and autophagy

Beyond the lipid metabolism-autophagy axis, another critical frontier in metabolic intervention lies in the reprogramming of amino acid metabolism. As previously stated, the metabolic pathway of glutamine is a viable novel therapeutic target because cancer cells are dependent on it 261. V-9302, the first small-molecule inhibitor that specifically targets the glutamine transporter alanine-serine-cysteine transporter 2 (ASCT2), has demonstrated strong anticancer potential in preclinical studies 262. This metabolic perturbation by inhibiting ASCT2-mediated glutamine uptake inhibits mTOR signaling, elevating protective autophagy and altering redox homeostasis to further promote cell death 262. Furthermore, the synergy observed when V-9302 and chloroquine are coupled emphasizes autophagy's dual function in this context and supports combination therapy techniques that regulate glutamine metabolism. Cancer cells use glutamine metabolism to fuel glycolysis, the TCA cycle, and biosynthesis, increasing chemoresistance. Glutaminase (GLS) is a crucial enzyme in this process, converting glutamine to glutamate and then α-ketoglutarate (α-KG), making it a promising therapeutic target 263. CB-839, a recognized GLS inhibitor, depletes TCA cycle intermediates, induces compensatory autophagy, and reduces proliferation 264, 265. CB-839 is particularly effective in glutamine-addicted tumors like TNBC, and it synergizes with paclitaxel to overcome resistance 266. Furthermore, combining CB-839 with the mTORC1 inhibitor AZD8055 simultaneously reduces glycolysis and glutamine metabolism, suggesting a viable therapy for glutamine-dependent malignancies 136, 267. Beyond CB-839, the glutamine antagonist prodrug JHU-083 suppresses tumor growth not only by impairing glutamine metabolism, which leads to mTOR inhibition and autophagy activation, but also by remodeling the tumor immune microenvironment, promoting CD8+ T cell activity while decreasing immunosuppressive myeloid cells 268, 269.

L-type amino acid transporter 1 (LAT1) mediates amino acid uptake, particularly leucine, and is typically overexpressed in malignancies such as NSCLC and PDAC, promoting mTOR signaling and tumor progression 270-272. Given this significance, JPH203, a specific LAT1 inhibitor, offers a promising treatment option. It reduces critical amino acid inflow, which inhibits mTOR activity and promotes autophagy 273. Preclinical evidence suggests its usefulness in reducing tumor growth in a variety of cancer types 274. Similarly, another LAT1 inhibitor, SKN103, slows cancer growth by blocking mTOR, activating autophagy, and inducing apoptosis. Moreover, its efficacy is enhanced in combination with cisplatin 275. Additionally, the flavonoid molecule baicalein, which comes from the traditional Chinese medicinal herb Scutellaria baicalensis, suppresses the levels of ASCT2, LAT1, and GLS1 protein expression, which limits glutamine metabolism 276, 277. This inhibition disrupts glutamine flux into the TCA cycle, reducing α-KG and ATP production, thereby impairing lung cancer cell proliferation 278. Simultaneously, baicalein suppresses mTOR signaling downstream of glutamine metabolism and activates autophagy, collectively promoting apoptotic cell death 276, 277. STAM-binding protein-like 1 (STAMBPL1), a deubiquitinase that enhances tumor progression and therapy resistance by modulating networks including mTOR, can influence the amino acid-sensing machinery central to this pathway 279. The natural compound Gamabufotalin (CS-6), derived from the traditional Chinese medicine Andrographis Paniculata, targets STAMBPL1 in HCC, inhibiting amino acid-related metabolic pathways, suppressing mTOR signaling, activating autophagy, and inducing apoptosis 280, 281. (Table 3).

Overall, focusing on autophagy and amino acid metabolism has become a very promising approach to cancer treatment. This strategy is particularly relevant for cancers harboring specific genetic alterations (e.g., Myc activation) or subtypes (e.g., TNBC), which develop a critical dependency on glutamine. As a result, key elements of the glutamine pathway, such as its primary transporters (like ASCT2 and LAT1) and the enzyme (like GLS), seem logical and attractive targets for intervention.

### 4.4 Small-molecule drugs targeting other metabolic processes and autophagy

In addition to the three classical metabolic pathways of glucose, lipid and amino acid metabolism, the interaction between nucleotide metabolism, mitochondrial quality control and autophagy has gradually become a research hotspot in the regulation of tumor metabolism. As discussed before, a bidirectional regulatory network exists between autophagy and nucleotide metabolism: autophagy replenishes the nucleotide pool through nucleic acid degradation, whereas the dynamic balance of nucleotide metabolism, together with RNA epigenetic modifications (e.g., m⁶A), in turn fine-tunes autophagic activity. Meanwhile, as the central hub of cellular metabolism, mitochondrial functional integrity is strictly controlled by mitophagy, and this quality control mechanism directly affects tumor cell survival and drug resistance under metabolic stress. Targeting this complex interactive network, a series of small-molecule compounds with novel mechanisms of action have emerged, providing new avenues for cancer therapy. (Table 4).

Given that polyamine levels are elevated in proliferating cells, polyamine biosynthesis has long been proposed as a potential therapeutic target in cancer 282. In this pathway, 5'-methylthioadenosine phosphorylase (MTAP) catalyzes the conversion of 5'-methylthioadenosine (MTA) into adenine and 5-methylthioribose-1-phosphate (MTR), which are subsequently converted into ATP and methionine, leading to the generation of S-adenosylmethionine (SAM), the principal methyl donor for metabolic and protein methylation reactions 283. Loss of MTAP is frequently observed in multiple cancers, including mesothelioma, urothelial carcinoma, pancreatic cancer, lung cancer, and glioma, rendering it an attractive target for synthetic lethality strategies 284, 285. Methylthio-DADMe-Immucillin-A (MTDIA), a highly potent transition-state inhibitor of MTAP, suppresses MTAP activity at nano- to picomolar concentrations. Studies in yeast models expressing human equilibrative nucleoside transporter 1 (hENT1) have shown that MTDIA not only disrupts the nucleotide salvage pathway but also enhances the activity of rapamycin, thereby effectively inducing autophagy and exerting broad antitumor activity in xenograft models of lung, prostate, colon, and TNBC 286. In terms of mitochondrial quality control, new research suggests that tumor cells maintain mitochondrial homeostasis via sphingolipid compensation and extracellular vesicle-mediated mitochondrial clearance. Eliglustat targets this mechanism by inhibiting glucosylceramide synthase, which promotes ceramide buildup within mitochondria, triggering compensatory mitophagy and eventually cancer cell death 22, 287. Sanggenol L (SL), a natural small chemical derived from mulberry, efficiently suppresses glioblastoma growth while considerably increasing sensitivity to the frontline chemotherapy drug temozolomide (TMZ) 288. SL upregulates the E3 ubiquitin ligase TRIM16, boosting ubiquitin-proteasome-dependent degradation of the essential autophagy receptor OPTN, therefore inhibiting mitophagic flow and triggering death 288. In contrast, the 3,4-diisobutyryl derivative of auxarthrol A (DAA), a fungal-derived small molecule, is an effective autophagy inducer. DAA disrupts the connection between dynein light chain 1 (LIC1) and RuvB-like AAA ATPase 1 (RUVBL1), causing ribosome collision and activating the GCN2-eIF2α-ATF4 axis. This initiates the integrated stress response (ISR), which results in autophagy-dependent cell death. DAA consistently demonstrates substantial anticancer activity in NSCLC and improves responsiveness to anti-PD-1 treatment 289. In addition, some recently synthesized drugs have demonstrated promise in targeting this regulatory network. Compound 33a, a new thioheterocyclic nucleoside derivative, has strong antiproliferative action in HCT116 colon cancer cells. Mechanistic studies reveal that 33a inhibits the c-MYC pathway, raises reactive oxygen species levels, and produces DNA damage, endoplasmic reticulum stress, and mitochondrial malfunction, while simultaneously suppressing autophagy 290. From a clinical translational standpoint, autophagy frequently plays a cytoprotective role during metabolic stress, implying that combining autophagy inducers and inhibitors may produce synergistic therapeutic effects. For example, the combination of MTDIA and chloroquine requires more exploration. Furthermore, specific manipulation of mitophagy (e.g., using drugs like SL) may allow for more precise therapeutic intervention while avoiding the systemic toxicity associated with global autophagy inhibition. Nonetheless, the majority of these drugs are still in the preclinical stage, and their pharmacokinetic properties, long-term toxicity, and efficacy across tumor types need to be thoroughly evaluated. Addressing these problems will be critical to moving from proof-of-concept to clinical translation.

In conclusion, targeting the interaction between nucleotide metabolism, mitochondrial quality control, and autophagy offers a promising avenue for therapeutic development. Small-molecule drugs that affect this network via various pathways have shown significant anticancer activity in preclinical trials, providing the groundwork for future clinical translation and the development of combination treatment regimens.

### 4.5 Clinical translation of autophagy-metabolism targeting strategies

While the preclinical evidence supporting autophagy-metabolism targeting is compelling, successful clinical translation remains limited. Here, we systematically summarize ongoing and completed clinical trials evaluating agents that modulate the autophagy-metabolism axis in cancer.

The growing body of preclinical evidence supporting autophagy-metabolism targeting has prompted extensive clinical evaluation of relevant agents. HCQ, the only FDA-approved autophagy inhibitor, has been extensively evaluated in clinical trials 29. A phase II trial (NCT01510119) combining HCQ with the mTOR inhibitor everolimus in renal cell carcinoma demonstrated manageable safety but limited efficacy, with only 14% partial response rate 291. However, the modest single-agent activity of HCQ has shifted clinical focus toward rational combination strategies. Preclinical models of PDAC indicate synergistic effects between MEK inhibition and autophagy signaling suppression 292. Several Phase I and II clinical trials are currently underway to further explore these combination therapies in PDAC patients. The Phase I THREAD trial (NCT03825289) and Phase II PaTcH trial (NCT05518110) both demonstrated the efficacy of trametinib combined with HCQ in treating patients with advanced pancreatic cancer 293. Furthermore, a Phase I clinical trial is examining the combination of the MEK inhibitor binimetinib and HCQ particularly for patients with KRAS-mutated pancreatic cancer (NCT04132505), highlighting the trend toward biomarker-driven patient selection 293.

Beyond autophagy inhibition, metabolic modulators that also affect autophagy have entered clinical testing. Metformin, a widely used drug for type 2 diabetes, has been repurposed for cancer therapy due to its ability to activate AMPK and induce autophagy 294. One of the largest current trials is NCT01101438, a three-phase, triple-masked randomized investigation comprising 3,649 participants. The study examines metformin as a supplement to traditional adjuvant treatment in early-stage breast cancer 295. A completed window-of-opportunity experiment in breast cancer patients (NCT01266486) found that metformin treatment stimulates AMPK phosphorylation and enhances autophagic flux in tumor tissues, giving direct evidence of on-target efficacy in people 296. Furthermore, a Phase IIa colorectal cancer prevention trial (NCT01312467) found that metformin significantly reduced adenoma recurrence rates in non-diabetic patients with elevated body mass index, providing clinical proof-of-concept for metabolic therapies in cancer prevention 204. Targeted metabolic inhibitors have recently made their way into the clinical setting. Phase II studies for the glutaminase inhibitor CB-839 have begun. CB-839 plus everolimus was assessed in patients with advanced renal cell cancer in the ENTRATA study (NCT03163667) 297. Exploratory analysis indicated benefit in patients with particular metabolic signatures, underscoring the significance of patient selection even though the combination did not meet its primary aim of enhancing progression-free survival. The potential of CB-839 in conjunction with cabozantinib for the treatment of metastatic renal cell carcinoma was further proven by the completed CANTATA trial (NCT03428217) 298. Significantly, correlative analyses from these trials showed that CB-839 treatment causes compensatory autophagy in patient tumors, highlighting the adaptive character of cancer metabolism and offering compelling evidence for the combination of autophagy blockers and glutaminase inhibitors 299.

All things considered, the aforementioned clinical experience offers important insights into the therapeutic potential of focusing on the autophagy-metabolism axis as well as the constraints of its applicability. The success of autophagy-targeting treatments is essentially dependent on the type of tumor, the stage of the disease, and the particular function of autophagy in each situation, as seen by the variation in results among various trials 300. While activating autophagy may trigger tumor suppression mechanisms in early-stage or residual disease, inhibiting autophagy may make established cancers more susceptible to chemotherapy by obstructing vital survival pathways 301. The intricate biological nature of autophagy throughout the cancer process is reflected in these conflicting tactics, which are not mutually exclusive. Thus, context-specific trial designs must replace a "one-size-fits-all" strategy in future research. Finding patient populations most likely to benefit from clinical trials based on tumor biology and illness stage should be the top objective. However, a significant shortcoming of existing approaches—the absence of accurate indicators to direct patient selection and therapy timing—is highlighted by the variation in therapeutic outcomes among research. Adaptive trial designs should be used to account for the dynamic nature of the autophagy response, and validated pharmacodynamic indicators should be included to demonstrate target engagement. Essentially, matching the right autophagy will be essential for effective clinical translation. One possible approach to overcoming metabolic plasticity and treatment resistance is to target autophagy in conjunction with metabolic therapies.

## 5. Conclusion and outlook

Autophagy and metabolic reprogramming are two unique traits of cancer cells that exhibit strong cross-regulation and are crucial adaptive mechanisms for tumor survival under stress. In order to show how cancer cells dynamically control energy and nutrient supply through autophagy, maintaining growth and developing drug resistance in nutrient-deficient microenvironments, this review offers a thorough investigation of their intricate interactions in important metabolic pathways (such as glucose, lipid, and amino acid metabolism) as well as in the emerging regulatory networks of nucleotide metabolism and mitochondrial metabolism. A novel therapeutic strategy is to target the autophagy-metabolism axis. Small-molecule medicines interfere with metabolic nodes and autophagy processes while also disrupting tumor bioenergetics and biosynthetic pathways, which eventually compromises the viability of cancer cells. This strategy has a great deal of promise to get over the current treatment constraints. Importantly, a key unresolved challenge is to delineate when autophagy should be inhibited versus activated in a tumor-specific and stage-dependent manner, which remains a major barrier to clinical translation.

In terms of treatment strategies, several small-molecule medications have shown synergistic modulation of metabolism and autophagy. For example, the lipid metabolism modulator MK8722 simultaneously influences autophagy flux and fatty acid synthesis by activating AMPK; the glucose metabolism inhibitor 2-DG combined with the autophagy inhibitor chloroquine blocks self-protective mechanisms of cancer cells under glucose deprivation; and the glutamine metabolism targeting CB-839 activates autophagy by inducing metabolic stress and blocking the mTOR pathway. These investigations offer theoretical support for combination therapy in addition to confirming the viability of the metabolism-autophagy axis as a therapeutic target. These preclinical findings have received additional support from emerging clinical research. As mentioned earlier, metformin's ability to promote autophagic flux has been confirmed in patient tumor tissues, and studies combining hydroxychloroquine with MEK inhibitors in KRAS-mutant pancreatic cancer have demonstrated a synergistic effect. The therapeutic significance of this adaptive response is highlighted by the compensatory autophagy observed following glutaminase inhibition with CB-839, providing strong support for the combination of metabolic inhibitors and autophagy blockers. When taken as a whole, these clinical results show that focusing on the autophagy-metabolism axis is not just a theoretical idea but also a workable method.

This field still faces a number of important obstacles despite tremendous advancements. Drug selection and therapy timing are complicated by autophagy's two-edged sword impact, which plays opposite functions at different stages of the tumor. In order to precisely determine treatment windows, it is necessary to develop imaging technologies or biomarkers (such LC3-II/p62 levels) that can monitor autophagy processes in real-time. Second, differences in metabolic requirements between cancer types and even within the same tumor are caused by tumor heterogeneity. Future initiatives should make use of technology such as single-cell sequencing to pinpoint metabolic weaknesses unique to subpopulations, directing tailored medication delivery. Furthermore, the need to strike a balance between safety and efficacy while creating new metabolic/autophagy modulators is highlighted by the removal of current medications like etomoxir from clinical use due to hepatotoxicity. Potential solutions include techniques like using nanocarriers to increase tumor-specific medication concentration. To better determine when and how autophagy should be targeted for cancer therapy, further research incorporating metabolic flux measurement, single-cell technologies, and *in vivo* tumor models will be crucial.

In summary, this study methodically investigates the relationships between autophagy and important metabolic pathways in cancer, suggesting therapeutic promise in focusing on their cross-regulatory processes. Although pharmaceutical approaches that target autophagy or cancer metabolism have demonstrated promising therapeutic potential, the context-dependent and occasionally contradictory roles of autophagy in tumor growth continue to challenge their clinical use. Therefore, it will be crucial to get a deeper mechanistic knowledge of how particular autophagy processes interact with metabolic networks across various tumor types. In the end, more accurate treatment approaches can be created by utilizing the autophagy-metabolism axis in cancer through future projects including merging metabolic analysis, functional genomics, and patient classification based on biomarkers.

## Figures and Tables

**Table 1:** Small-molecule drugs targeting the glycolytic pathway and autophagy

Drug	Chemical Structure	Targets/Pathways	Mechanisms of action	Effect on autophagy	Cancer	Ref
2-DG	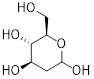	HK2	2-DG triggers glucose deprivation, activates AMPK, and increases ROS in cancer cells.	Activate	Liver, Breast, Lung cancer	190
Metformin	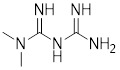	AMPK	By inhibiting hepatic gluconeogenesis, lowering cellular energy levels, and activating AMPK, thereby inhibiting the mTOR pathway.	Activate	Pancreatic, Colon, Breast cancer	200
Berberine	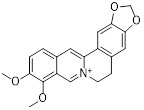	AMPK/mTOR/ULK1	Inhibition of the AMPK/mTOR/ULK1 pathway weakens glycolysis-dependent energy production and induces mitochondrial dysfunction.	Activate	GBM	211
Compound 3K	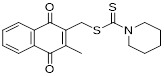	Akt/AMPK/mTOR	Disrupting glycolysis by regulating the Akt/AMPK/mTOR pathway	Activate	Ovarian cancer	212
3-BrPA	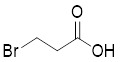	HK2	Inhibiting HK2 activity, inhibiting glycolysis	Activate	Myeloma	194
Polymethoxylated flavones (PMFs)	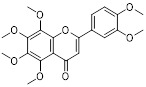	Aerobic glycolysis-ROS-autophagy signaling axis	Interfering with aerobic glycolysis in tumor cells reduces ROS production.	Inhibit	Colon cancer	216
STF-31	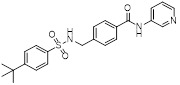	GLUT1	Reduce glucose intake and activate the AMPK pathway	Activate	Thyroid cancer	221
BAY-876	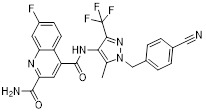	GLUT1	Reduce glucose intake and activate the AMPK pathway	Activate	Thyroid cancer	221
Spautin-1	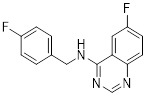	USP10 and USP13	Inhibition of the deubiquitinating activity of USP10 and USP13	Inhibit	Prostate cancer	226
SBI-0206965	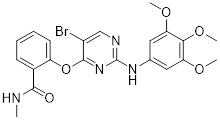	ULK1	Inhibition of autophagy and PPP flux triggers apoptosis	Inhibit	Clear Cell Renal Carcinoma	232

**Table 2:** Small-molecule drugs targeting lipid metabolism and autophagy

Drug	Chemical Structure	Targets/Pathways	Mechanisms of action	Effect on autophagy		Cancer	Ref
Rottlerin (Rott)	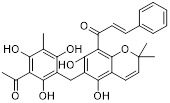	PI3K/Akt/mTOR	Inhibits mTORC1, triggering autophagy		Activate	Prostate cancer	234
Dehydrocostus lactone	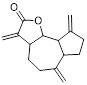	ACLY	Inhibits ACLY, reduces fatty acid synthesis, down-regulates LAMP1 and LAMP2 expression, and hinders protective autophagy		Inhibit	Stomach cancer	239
MK8722 (C_24_H_20_ClN_3_O_4_)	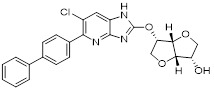	FASN	MK8722 activates early autophagy but inhibits late autophagy by inducing FASN-dependent reprogramming of lipid metabolism		Early activation; late inhibition	EOC	243
PFK158	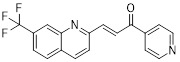	PFKFB3	Activates autophagy and promotes apoptosis; also targets glycolytic and adipogenic pathways to inhibit tumor growth		Activate	Ovarian, Cervical cancer	251
Schisandrin B	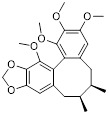	AMPK/mTOR	Sch B inhibits hepatic steatosis and activates autophagy through the AMPK/mTOR pathway to promote FAO		Activate	Colorectal cancer	236
LYN-1604	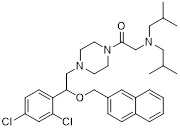	ULK1	ULK1 agonists inhibit lipid synthesis by regulating lipid metabolism (e.g., promoting fatty acid oxidation).		Activate	Breast, Pancreatic cancer	248
Resveratrol	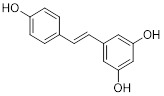	SREBP1	Inhibiting SREBP1-mediated cell survival signaling by regulating SREBP1 expression to suppress lipid metabolism		Activate	Liver, Pancreas, Colorectal cancer	260
Etomoxir	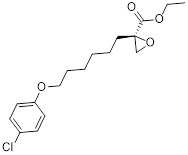	CPT1	Irreversibly inhibits the activity of CPT1, blocking the transport of fatty acids into mitochondria.		Activate	Ovary cancer	252
CPI-613	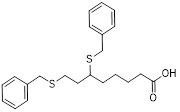	AMPK-ACC	Inhibition of lipid metabolism through activation of AMPK signaling, triggering autophagy and ACC inhibition		Activate	Pancreatic cancer	246
							

**Table 3:** Small-molecule drugs targeting amino acids metabolism and autophagy

Drug	Chemical Structure	Targets/Pathways	Mechanisms of action	Effect on autophagy	Cancer	Ref
V-9302	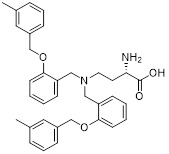	ASCT2	Competitively inhibits glutamine uptake mediated by ASCT2, suppresses mTOR signaling pathways, and alters intracellular redox status	Activate	HCC	262
CB-839	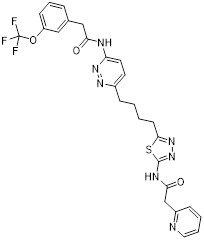	Glutaminase	Inhibition of glutaminase by CB-839 results in decreased alpha-ketoglutarate production, TCA cycle starvation, and induction of compensatory autophagy in cancer cells.	Activate	Breast cancer	136, 264
JHU-083	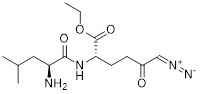	Glutamine metabolism	Blocking glutamine metabolism, reducing mTOR signaling, and activating autophagy	Activate	Neuroglioma	268
JPH203	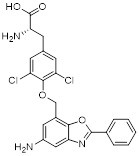	LAT1	Reduced uptake of leucine and other amino acids inhibits mTOR activity and leads to activation of autophagy	Activate	Lung, pancreatic cancer	274
SKN103	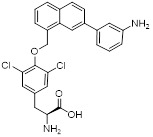	LAT1	Inhibits leucine transport, downregulates mTOR signaling, and activates autophagy	Activate	Lung, pancreatic cancer	275
Baicalein	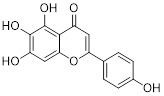	ASCT2, LAT1, GLS1	Inhibits the protein expression levels of ASCT2, LAT1, and GLS1, thereby suppressing glutamine metabolism; inhibits the mTOR metabolic pathway.	Activate	Lung cancer	276, 277
Gamabufotalin (CS-6)	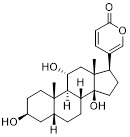	STAMBPL1	Inhibits HCC malignancy by down-regulating STAMBPL1, inhibiting amino acid-related metabolic pathways, suppressing mTOR activity, stimulating autophagy, and leading to increased apoptosis.	Activate	Liver cancer	281

**Table 4:** Small-molecule drugs targeting other metabolic processes and autophagy

Drug	Chemical Structure	Targets/Pathways	Mechanisms of action	Effect on autophagy	Cancer	Ref
Methylthio-DADMe-Immucillin-A (MTDIA)	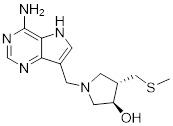	MTAP	MTDIA disrupts the nucleotide salvage pathway, enhances the activity of rapamycin, and thereby effectively induces autophagy	Activate	Lung, prostate, colorectal cancer, and TNBC	286
Eliglustat	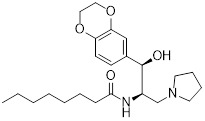	Glucosylceramide synthase	Inhibiting glucose-neuraminidase promotes the accumulation of neuraminidase in mitochondria, triggering compensatory mitochondrial autophagy, which ultimately leads to cancer cell death	Activate	TNBC	22, 287
Sanggenol L (SL)	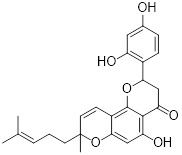	TRIM16	SL upregulates the E3 ubiquitin ligase TRIM16, which degrades the key autophagy receptor OPTN, thereby blocking the mitochondrial autophagy flux and inducing apoptosis	Inhibit	Glioblastoma	288
3,4-diisobutyryl derivative of auxarthrol A (DAA)	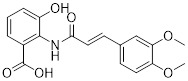	LIC1	DAA acts directly on LIC1, disrupting its interaction with RUVBL1 and activating the GCN2-eIF2α-ATF4 axis	Activate	NSCLC	289
Compound 33a	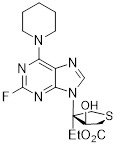	c-MYC pathway	Increases reactive oxygen species levels and induces DNA damage, endoplasmic reticulum stress, and mitochondrial dysfunction	Inhibit	Colon cancer	290

## Abbreviations

2-DG: 2-deoxy-D-glucose
3-BrPA: 3-Bromopyruvic acid
α-KG: α-ketoglutarate
Acetyl-CoA: Acetyl-CoAAcetyl-coenzyme A
ACC: Acetyl-CoA carboxylase
ACLY: ATP citrate lyase
ADIPOR1: Adiponectin receptor 1
Agrp: Agouti-related peptide
Akt: Protein kinase B
ALKBH5: AlkB homolog 5
AML: Acute Myeloid Leukemia
AMPK: Adenosine monophosphate-activated protein kinase
ASCT2: Alanine-serine-cysteine transporter 2
ATF4: Activating transcription factor 4
ATG: Autophagy-related genes
ATG4A: Autophagy-related 4 homolog A
ATGL: Adipose triglyceride lipase
ATP: Adenosine triphosphate
BCAAs: Branched-chain amino acids
BCAT1: Branched-chain amino acids transaminase isoenzyme 1
BCAT2: Branched-chain amino acids transaminase isoenzyme 2
BC-CoA: Branched-chain acyl-CoA
BCKA: Branched-chain ketone acid
BCKDH: Branched-chain α-ketoacid dehydrogenase complex
Bcl-2: B-cell lymphoma-2
CAF: Cancer-associated fibroblasts
CCL7: Chemokine (C-C motif) ligand 7
ccRCC: Clear cell renal cell carcinoma
CD36: Cluster of differentiation 36
CMA: Chaperone-mediated autophagy
c-MYC: Cellular myelocytomatosis oncogene
CPT1: Carnitine palmitoyltransferase 1
CQ: Chloroquine
CSCs: Cancer stem cells‌
CXCL10: C-X-C motif chemokine ligand 10
CXCL12: C-X-C motif chemokine ligand 12
Dehy: Dehydrocostus lactone
DAA: 3,4-diisobutyryl derivative of auxarthrol A
dNTPs: deoxyribonucleotide counterparts
eIF2α: Eukaryotic initiation factor 2α
EMT: Epithelial-mesenchymal transition
EOC: Epithelial ovarian cancer
ERRα: Estrogen-related receptor α
F-2,6-BP: Fructose-2,6-bisphosphate
FABP4: Fatty acids binding protein-4
FAO: Fatty acids oxidation
FASN: Fatty acid synthase
FFAs: Free fatty acids
FH: Fumarate hydratase
FIP200: Focal adhesion kinase family interacting protein of 200 kDa
GATOR: Gap activity towards rags
GBM: Glioblastoma multiforme
GC: Gastric cancer
GCN2: General control non-derepressible 2
GLS: Glutaminase
GLUD: Glutamate dehydrogenase
GLUT1: Glucose transporter protein 1
GSH: Glutathione
HCQ: Hydroxychloroquine
HCC: Hepatocellular carcinoma
HER2: Human Epidermal Growth Factor Receptor 2
hENT1: Human equilibrative nucleoside transporter 1
HK2: Hexokinase 2
HIF1: Hypoxia-inducible factor 1
HMGCS1: Hydroxymethylglutaryl-CoA synthase 1
HSC70: Heat shock cognate protein of 70 kDa
IDH: Isocitrate dehydrogenase
IKKβ: IκB kinase β
IL-6: Interleukin-6
IMP: Inosine monophosphate
ISR: Integrated stress response
JNK: c- N-terminal kinase
LAMP2A: Lysosome-associated membrane protein 2A
LAT1: L-type amino acid transporter 1
LC3: Light chain-3
LDHB: Lactate dehydrogenase B
LDs: Lipid droplets
LIC1: Light chain 1
m⁶A: N6-methyladenosine
MACC1: Metastasis-associated in colon cancer 1
MCTs: Monocarboxylate transporters
MTA: 5'-methylthioadenosine
MTAP: 5'-methylthioadenosine phosphorylase
MTDIA: Methylthio-DADMe-Immucillin-A
mTOR: Mammalian target of rapamycin
mTORC1: Mammalian targets of rapamycin complex 1
MTR: 5-methylthioribose-1-phosphate
NADH: Nicotinamide adenine dinucleotide
NAFLD: Non-alcoholic fatty liver disease
NF-κB: Nuclear Factor kappa-B
NLR: NOD-like receptor
NLRX1: NOD-like receptor family member X1
NMN: Nicotinamide mononucleotide
NMPs: Nucleoside monophosphates
Nrf2: Nuclear Factor Erythroid 2-Related Factor 2
NSCLC: Non-small cell lung cancer
NTPs: Nucleotide triphosphates
OXPHOS: Oxidative phosphorylation
p53: Tumor protein 53
PDAC: Pancreatic Ductal Adenocarcinoma
PFKFB3: Fructose-6-phosphate-2-kinase/fructose-2,6-bisphosphatase 3 enzyme
PI3K: Phosphatidylinositol 3-kinase
PI3KC3: Phosphatidylinositol 3-kinase class III
PKR: Protein kinase R
PLIN2: Perilipin 2
PLIN3: Perilipin 3
PLINs: Perilipins
PMFs: Polymethoxyflavonoids
PPARγ: Peroxisome proliferator-activated receptor γ
PTEN: Phosphatase and tensin homolog
PDK1: Pyruvate dehydrogenase kinase 1
PET: Positron emission tomography
PKM2: Pyruvate kinase M2
PPP: Pentose phosphate pathway
PRPP: Phosphoribosyl pyrophosphate
Rag A/B: Recombination-activating gene A/B
Rag C/D: Recombination-activating gene C/D
Rag GTPases: Ras-related GTP-binding proteins
Rheb: Ras homolog enriched in brain
ROS: Reactive oxygen species
Rott: Rottlerin
RUVBL1: RuvB-like AAA ATPase 1
SAM: S-adenosylmethionine
SCD1: Stearoyl-CoA desaturase 1
Sch B: Schisandrin B
SDH: Succinate dehydrogenase
SQSTM1/p62: Sequestosome 1
SL: Sanggenol L
SLC1A5: Solute carrier family 1, member 5
SNAT2: Sodium-coupled neutral amino acid transporter 2
SREBP1: Sterol regulatory element-binding protein 1
STAMBPL1: STAM-binding protein-like 1
TCA: Tricarboxylic acid cycle
TECs: Tumor endothelial cells
TFEB: Transcription factor EB
TGFβs: Transforming Growth Factor-β Superfamily
TME: Tumor microenvironment
TNBC: Triple-negative breast cancer
TSC2: Tuberous Sclerosis 2
ULK1: Unc-51-like kinase 1
UMP: Uridine monophosphate
UPR: Unfolded protein response
USP10: Ubiquitin-specific peptidase 10
VEGFA: Vascular endothelial growth factor
VMP1: Vacuolar membrane protein 1
VPS34: Vacuolar Protein Sorting 34

## References

[1] Levine B, Klionsky DJ (2004). Development by self-digestion: molecular mechanisms and biological functions of autophagy. Dev Cell.

[2] White E, Mehnert JM, Chan CS (2015). Autophagy, Metabolism, and Cancer. Clin Cancer Res.

[3] Liu S, Yao S, Yang H, Liu S, Wang Y (2023). Autophagy: Regulator of cell death. Cell Death & Disease.

[4] Barnard RA, Regan DP, Hansen RJ, Maycotte P, Thorburn A, Gustafson DL (2016). Autophagy Inhibition Delays Early but Not Late-Stage Metastatic Disease. J Pharmacol Exp Ther.

[5] Levine B, Kroemer G (2008). Autophagy in the pathogenesis of disease. Cell.

[6] Yamamoto K, Venida A, Yano J, Biancur DE, Kakiuchi M, Gupta S (2020). Autophagy promotes immune evasion of pancreatic cancer by degrading MHC-I. Nature.

[7] Judge A, Dodd MS (2020). Metabolism. Essays Biochem.

[8] Mathew R, White E (2011). Autophagy, stress, and cancer metabolism: what doesn't kill you makes you stronger. Cold Spring Harb Symp Quant Biol.

[9] Rabinowitz JD, White E (2010). Autophagy and metabolism. Science.

[10] Park JH, Pyun WY, Park HW (2020). Cancer metabolism: Phenotype, signaling and therapeutic targets. Cells.

[11] Ferro F, Servais S, Besson P, Roger S, Dumas JF, Brisson L (2020). Autophagy and mitophagy in cancer metabolic remodelling. Semin Cell Dev Biol.

[12] Yang J, Zhou R, Ma Z (2019). Autophagy and energy metabolism. Adv Exp Med Biol.

[13] Fu Y, Zhang J, Qin R, Ren Y, Zhou T, Han B (2025). Activating autophagy to eliminate toxic protein aggregates with small molecules in neurodegenerative diseases. Pharmacol Rev.

[14] Mizushima N (2009). Physiological functions of autophagy. Curr Top Microbiol Immunol.

[15] Adelipour M, Saleth LR, Ghavami S, Alagarsamy KN, Dhingra S, Allameh A (2022). The role of autophagy in the metabolism and differentiation of stem cells. Biochim Biophys Acta Mol Basis Dis.

[16] Glick D, Barth S, Macleod KF (2010). Autophagy: cellular and molecular mechanisms. J Pathol.

[17] Miller DR, Thorburn A (2021). Autophagy and organelle homeostasis in cancer. Dev Cell.

[18] Nishimura T, Tooze SA (2020). Emerging roles of ATG proteins and membrane lipids in autophagosome formation. Cell Discov.

[19] Kocaturk NM, Akkoc Y, Kig C, Bayraktar O, Gozuacik D, Kutlu O (2019). Autophagy as a molecular target for cancer treatment. Eur J Pharm Sci.

[20] Chen YF, Liu H, Luo XJ, Zhao Z, Zou ZY, Li J (2017). The roles of reactive oxygen species (ROS) and autophagy in the survival and death of leukemia cells. Crit Rev Oncol Hematol.

[21] Galluzzi L, Pietrocola F, Bravo-San Pedro JM, Amaravadi RK, Baehrecke EH, Cecconi F (2015). Autophagy in malignant transformation and cancer progression. Embo j.

[22] Lu Y, Li Z, Zhang S, Zhang T, Liu Y, Zhang L (2023). Cellular mitophagy: Mechanism, roles in diseases and small molecule pharmacological regulation. Theranostics.

[23] Zhang S, Peng X, Yang S, Li X, Huang M, Wei S (2022). The regulation, function, and role of lipophagy, a form of selective autophagy, in metabolic disorders. Cell Death Dis.

[24] Subramani S, Malhotra V (2013). Non-autophagic roles of autophagy-related proteins. EMBO Rep.

[25] Mizushima N (2005). The pleiotropic role of autophagy: from protein metabolism to bactericide. Cell Death Differ.

[26] Kundu M, Thompson CB (2008). Autophagy: basic principles and relevance to disease. Annu Rev Pathol.

[27] Eskelinen EL (2011). The dual role of autophagy in cancer. Curr Opin Pharmacol.

[28] Singh SS, Vats S, Chia AY, Tan TZ, Deng S, Ong MS (2018). Dual role of autophagy in hallmarks of cancer. Oncogene.

[29] Onorati AV, Dyczynski M, Ojha R, Amaravadi RK (2018). Targeting autophagy in cancer. Cancer.

[30] Dou Z, Xu C, Donahue G, Shimi T, Pan JA, Zhu J (2015). Autophagy mediates degradation of nuclear lamina. Nature.

[31] Yan RL, Chen RH (2022). Autophagy and cancer metabolism-The two-way interplay. IUBMB Life.

[32] Hao M, Yeo SK, Turner K, Harold A, Yang Y, Zhang X (2021). Autophagy blockade limits HER2+ breast cancer tumorigenesis by perturbing HER2 trafficking and promoting release via small extracellular vesicles. Dev Cell.

[33] Liang P, Li Z, Chen Z, Chen Z, He F, Jin T (2025). HER2 regulates autophagy and promotes migration in gastric cancer cells through the cGAS-STING pathway. Anticancer Drugs.

[34] Levine B, Kroemer G (2019). Biological functions of autophagy genes: A disease perspective. Cell.

[35] Inami Y, Waguri S, Sakamoto A, Kouno T, Nakada K, Hino O (2011). Persistent activation of Nrf2 through p62 in hepatocellular carcinoma cells. J Cell Biol.

[36] Dong W, Wan J, Yu H, Shen B, Yang G, Nie Q (2023). Nrf2 protects against methamphetamine-induced nephrotoxicity by mitigating oxidative stress and autophagy in mice. Toxicol Lett.

[37] Debnath J, Gammoh N, Ryan KM (2023). Autophagy and autophagy-related pathways in cancer. Nature Reviews Molecular Cell Biology.

[38] Diakos CI, Charles KA, McMillan DC, Clarke SJ (2014). Cancer-related inflammation and treatment effectiveness. Lancet Oncol.

[39] Balkwill FR, Mantovani A (2012). Cancer-related inflammation: common themes and therapeutic opportunities. Semin Cancer Biol.

[40] Gong C, Bauvy C, Tonelli G, Yue W, Deloménie C, Nicolas V (2013). Beclin 1 and autophagy are required for the tumorigenicity of breast cancer stem-like/progenitor cells. Oncogene.

[41] Wolf J, Dewi DL, Fredebohm J, Müller-Decker K, Flechtenmacher C, Hoheisel JD (2013). A mammosphere formation RNAi screen reveals that ATG4A promotes a breast cancer stem-like phenotype. Breast Cancer Res.

[42] Zhang L, Li J, Ouyang L, Liu B, Cheng Y (2016). Unraveling the roles of Atg4 proteases from autophagy modulation to targeted cancer therapy. Cancer Lett.

[43] Mukhopadhyay S, Mahapatra KK, Praharaj PP, Patil S, Bhutia SK (2022). Recent progress of autophagy signaling in tumor microenvironment and its targeting for possible cancer therapeutics. Semin Cancer Biol.

[44] Sahai E, Astsaturov I, Cukierman E, DeNardo DG, Egeblad M, Evans RM (2020). A framework for advancing our understanding of cancer-associated fibroblasts. Nat Rev Cancer.

[45] LeBleu VS, Kalluri R (2018). A peek into cancer-associated fibroblasts: origins, functions and translational impact. Dis Model Mech.

[46] Steeg PS (2016). Targeting metastasis. Nat Rev Cancer.

[47] Yuan Z, He J, Li Z, Fan B, Zhang L, Man X (2024). Targeting autophagy in urological system cancers: From underlying mechanisms to therapeutic implications. Biochim Biophys Acta Rev Cancer.

[48] Yun CW, Lee SH (2018). The Roles of Autophagy in Cancer. Int J Mol Sci.

[49] Hamurcu Z, Delibaşı N, Geçene S, Şener EF, Dönmez-Altuntaş H, Özkul Y (2018). Targeting LC3 and Beclin-1 autophagy genes suppresses proliferation, survival, migration and invasion by inhibition of Cyclin-D1 and uPAR/Integrin β1/ Src signaling in triple negative breast cancer cells. J Cancer Res Clin Oncol.

[50] Liu H, He Z, von Rütte T, Yousefi S, Hunger RE, Simon HU (2013). Down-regulation of autophagy-related protein 5 (ATG5) contributes to the pathogenesis of early-stage cutaneous melanoma. Sci Transl Med.

[51] Hashimoto I, Koizumi K, Tatematsu M, Minami T, Cho S, Takeno N (2008). Blocking on the CXCR4/mTOR signalling pathway induces the anti-metastatic properties and autophagic cell death in peritoneal disseminated gastric cancer cells. Eur J Cancer.

[52] Gundamaraju R, Lu W, Paul MK, Jha NK, Gupta PK, Ojha S (2022). Autophagy and EMT in cancer and metastasis: Who controls whom?. Biochim Biophys Acta Mol Basis Dis.

[53] Peng YF, Shi YH, Ding ZB, Ke AW, Gu CY, Hui B (2013). Autophagy inhibition suppresses pulmonary metastasis of HCC in mice via impairing anoikis resistance and colonization of HCC cells. Autophagy.

[54] Chen G, Gao C, Jiang S, Cai Q, Li R, Sun Q (2024). Fusobacterium nucleatum outer membrane vesicles activate autophagy to promote oral cancer metastasis. J Adv Res.

[55] Huang Y, Hong W, Wei X (2022). The molecular mechanisms and therapeutic strategies of EMT in tumor progression and metastasis. J Hematol Oncol.

[56] Guo Z, Ashrafizadeh M, Zhang W, Zou R, Sethi G, Zhang X (2024). Molecular profile of metastasis, cell plasticity and EMT in pancreatic cancer: a pre-clinical connection to aggressiveness and drug resistance. Cancer Metastasis Rev.

[57] Xue W, Yang L, Chen C, Ashrafizadeh M, Tian Y, Sun R (2024). Wnt/β-catenin-driven EMT regulation in human cancers. Cell Mol Life Sci.

[58] Wu Q, Yang Z, Nie Y, Shi Y, Fan D (2014). Multi-drug resistance in cancer chemotherapeutics: mechanisms and lab approaches. Cancer Lett.

[59] Zhang X, Zhu C, Huang B, Wang H (2025). The dual-edged sword: AlkB homolog 5-mediated autophagy regulation in cancers - molecular mechanisms and therapeutic implications: A review. Int J Biol Macromol.

[60] Guo JY, Karsli-Uzunbas G, Mathew R, Aisner SC, Kamphorst JJ, Strohecker AM (2013). Autophagy suppresses progression of K-ras-induced lung tumors to oncocytomas and maintains lipid homeostasis. Genes Dev.

[61] Kubik J, Humeniuk E, Adamczuk G, Madej-Czerwonka B, Korga-Plewko A (2022). Targeting Energy Metabolism in Cancer Treatment. Int J Mol Sci.

[62] Goldsmith J, Levine B, Debnath J (2014). Autophagy and cancer metabolism. Methods Enzymol.

[63] Hanahan D (2022). Hallmarks of Cancer: New Dimensions. Cancer Discov.

[64] Qin Y, Ashrafizadeh M, Mongiardini V, Grimaldi B, Crea F, Rietdorf K (2023). Autophagy and cancer drug resistance in dialogue: Pre-clinical and clinical evidence. Cancer Lett.

[65] Wang X, Zhou L, Wang H, Chen W, Jiang L, Ming G (2023). Metabolic reprogramming, autophagy, and ferroptosis: Novel arsenals to overcome immunotherapy resistance in gastrointestinal cancer. Cancer Med.

[66] Lin L, Huang H, Liao W, Ma H, Liu J, Wang L (2015). MACC1 supports human gastric cancer growth under metabolic stress by enhancing the Warburg effect. Oncogene.

[67] Zhao L, Liu Y, Zhang S, Wei L, Cheng H, Wang J (2022). Impacts and mechanisms of metabolic reprogramming of tumor microenvironment for immunotherapy in gastric cancer. Cell Death Dis.

[68] Du W, Xu A, Huang Y, Cao J, Zhu H, Yang B (2021). The role of autophagy in targeted therapy for acute myeloid leukemia. Autophagy.

[69] Yang S, Sun Y, Guo Y, Zhao Z, Hu F, Cong L (2024). The glycolysis-related AMPK/ULK signaling pathway mediates the inhibitory effect of adiponectin in prostate cancer cells. Mol Cell Endocrinol.

[70] Mukha A, Kahya U, Dubrovska A (2021). Targeting glutamine metabolism and autophagy: the combination for prostate cancer radiosensitization. Autophagy.

[71] Ikeda S, Abe F, Matsuda Y, Kitadate A, Takahashi N, Tagawa H (2020). Hypoxia-inducible hexokinase-2 enhances anti-apoptotic function via activating autophagy in multiple myeloma. Cancer Sci.

[72] Paul S, Ghosh S, Kumar S (2022). Tumor glycolysis, an essential sweet tooth of tumor cells. Semin Cancer Biol.

[73] Fukushi A, Kim HD, Chang YC, Kim CH (2022). Revisited Metabolic Control and Reprogramming Cancers by Means of the Warburg Effect in Tumor Cells. Int J Mol Sci.

[74] Marcucci F, Rumio C (2023). On the Role of Glycolysis in Early Tumorigenesis-Permissive and Executioner Effects. Cells.

[75] Gatenby RA, Gillies RJ (2004). Why do cancers have high aerobic glycolysis?. Nat Rev Cancer.

[76] Wu W, Zhao S (2013). Metabolic changes in cancer: beyond the Warburg effect. Acta Biochim Biophys Sin (Shanghai).

[77] King KE, Losier TT, Russell RC (2021). Regulation of Autophagy Enzymes by Nutrient Signaling. Trends Biochem Sci.

[78] Roberts DJ, Tan-Sah VP, Ding EY, Smith JM, Miyamoto S (2014). Hexokinase-II positively regulates glucose starvation-induced autophagy through TORC1 inhibition. Mol Cell.

[79] Ke R, Xu Q, Li C, Luo L, Huang D (2018). Mechanisms of AMPK in the maintenance of ATP balance during energy metabolism. Cell Biol Int.

[80] Wang X, Li S, Lin S, Han Y, Zhan T, Huang Z (2025). Oncogenic RAS induces a distinctive form of non-canonical autophagy mediated by the P38-ULK1-PI4KB axis. Cell Res.

[81] Pangilinan C, Klionsky DJ, Liang C (2024). Emerging dimensions of autophagy in melanoma. Autophagy.

[82] Jalouli M (2025). Emerging Role of Hypoxia-Inducible Factors (HIFs) in Modulating autophagy: perspectives on cancer therapy. Int J Mol Sci.

[83] Xie X, Zhang Y, Li Y, Guo N, Guo X, Wang F (2025). Deubiquitinase OTUD4 stabilizes SLC5A2 to promote pancreatic cancer proliferation and migration through enchaining glycolysis-mediated autophagy. Faseb j.

[84] Raj S, Chandel V, Kumar A, Kesari KK, Asthana S, Ruokolainen J (2020). Molecular mechanisms of interplay between autophagy and metabolism in cancer. Life Sci.

[85] Ye J, Zhang J, Zhu Y, Wang L, Jiang X, Liu B (2023). Targeting autophagy and beyond: Deconvoluting the complexity of Beclin-1 from biological function to cancer therapy. Acta Pharm Sin B.

[86] Wang L, Yang L, Yang Z, Tang Y, Tao Y, Zhan Q (2019). Glycolytic enzyme PKM2 mediates autophagic activation to promote cell survival in NPM1-mutated leukemia. Int J Biol Sci.

[87] Mao X, Huang L, Liu X, Lin X, Wu Q, Wang X (2025). High glucose levels promote glycolysis and cholesterol synthesis via ERRα and suppress the autophagy-lysosomal pathway in endometrial cancer. Cell Death Dis.

[88] Huang J, Zhao X, Li X, Peng J, Yang W, Mi S (2021). HMGCR inhibition stabilizes the glycolytic enzyme PKM2 to support the growth of renal cell carcinoma. PLoS Biol.

[89] Qin L, Tian Y, Yu Z, Shi D, Wang J, Zhang C (2016). Targeting PDK1 with dichloroacetophenone to inhibit acute myeloid leukemia (AML) cell growth. Oncotarget.

[90] Zou L, Liao M, Zhen Y, Zhu S, Chen X, Zhang J (2022). Autophagy and beyond: Unraveling the complexity of UNC-51-like kinase 1 (ULK1) from biological functions to therapeutic implications. Acta Pharm Sin B.

[91] Singh M, Afonso J, Sharma D, Gupta R, Kumar V, Rani R (2023). Targeting monocarboxylate transporters (MCTs) in cancer: How close are we to the clinics?. Semin Cancer Biol.

[92] Dhup S, Dadhich RK, Porporato PE, Sonveaux P (2012). Multiple biological activities of lactic acid in cancer: influences on tumor growth, angiogenesis and metastasis. Curr Pharm Des.

[93] Brisson L, Bański P, Sboarina M, Dethier C, Danhier P, Fontenille MJ (2016). Lactate dehydrogenase B controls lysosome activity and autophagy in cancer. Cancer Cell.

[94] Doherty JR, Cleveland JL (2013). Targeting lactate metabolism for cancer therapeutics. J Clin Invest.

[95] Vaupel P, Multhoff G (2021). Revisiting the Warburg effect: historical dogma versus current understanding. J Physiol.

[96] Wang Y, Stancliffe E, Fowle-Grider R, Wang R, Wang C, Schwaiger-Haber M (2022). Saturation of the mitochondrial NADH shuttles drives aerobic glycolysis in proliferating cells. Mol Cell.

[97] Vaarwerk B, Breunis WB, Haveman LM, de Keizer B, Jehanno N, Borgwardt L (2021). Fluorine-18-fluorodeoxyglucose (FDG) positron emission tomography (PET) computed tomography (CT) for the detection of bone, lung, and lymph node metastases in rhabdomyosarcoma. Cochrane Database Syst Rev.

[98] Wei J, Huang K, Chen Z, Hu M, Bai Y, Lin S (2020). Characterization of glycolysis-associated molecules in the tumor microenvironment revealed by pan-cancer tissues and lung cancer single cell data. Cancers (Basel).

[99] Röhrig F, Schulze A (2016). The multifaceted roles of fatty acid synthesis in cancer. Nat Rev Cancer.

[100] Carracedo A, Cantley LC, Pandolfi PP (2013). Cancer metabolism: fatty acid oxidation in the limelight. Nat Rev Cancer.

[101] Shin DW (2020). Lipophagy: Molecular mechanisms and implications in metabolic disorders. Mol Cells.

[102] Boroughs LK, DeBerardinis RJ (2015). Metabolic pathways promoting cancer cell survival and growth. Nat Cell Biol.

[103] Park S, Oh TS, Kim S, Kim EK (2019). Palmitate-induced autophagy liberates monounsaturated fatty acids and increases Agrp expression in hypothalamic cells. Anim Cells Syst (Seoul).

[104] Mei S, Ni HM, Manley S, Bockus A, Kassel KM, Luyendyk JP (2011). Differential roles of unsaturated and saturated fatty acids on autophagy and apoptosis in hepatocytes. J Pharmacol Exp Ther.

[105] Zaytseva YY, Harris JW, Mitov MI, Kim JT, Butterfield DA, Lee EY (2015). Increased expression of fatty acid synthase provides a survival advantage to colorectal cancer cells via upregulation of cellular respiration. Oncotarget.

[106] Zaugg K, Yao Y, Reilly PT, Kannan K, Kiarash R, Mason J (2011). Carnitine palmitoyltransferase 1C promotes cell survival and tumor growth under conditions of metabolic stress. Genes Dev.

[107] Khaliq AM, Rajamohan M, Saeed O, Mansouri K, Adil A, Zhang C (2024). Spatial transcriptomic analysis of primary and metastatic pancreatic cancers highlights tumor microenvironmental heterogeneity. Nat Genet.

[108] Shi C, Zhang Z, Xu R, Zhang Y, Wang Z (2023). Contribution of HIF-1α/BNIP3-mediated autophagy to lipid accumulation during irinotecan-induced liver injury. Sci Rep.

[109] Yang P, Qin H, Li Y, Xiao A, Zheng E, Zeng H (2022). CD36-mediated metabolic crosstalk between tumor cells and macrophages affects liver metastasis. Nat Commun.

[110] Gao S, van der Veen S (2024). Gonococcal OMVs induce epithelial cell mitophagy in a dual PorB-dependent manner to enhance intracellular survival. Autophagy.

[111] Hama Y, Morishita H, Mizushima N (2022). Regulation of ER-derived membrane dynamics by the DedA domain-containing proteins VMP1 and TMEM41B. EMBO Rep.

[112] Ascenzi F, De Vitis C, Maugeri-Saccà M, Napoli C, Ciliberto G, Mancini R (2021). SCD1, autophagy and cancer: implications for therapy. J Exp Clin Cancer Res.

[113] Chen Y, Chen J, Zou Z, Xu L, Li J (2024). Crosstalk between autophagy and metabolism: implications for cell survival in acute myeloid leukemia. Cell Death Discov.

[114] Li X, Zhang J, Luo S, Yu X, Song C (2025). Crosstalk between lipid droplets and autophagy in cancer: A nexus for therapeutic targeting. Pharmacol Res.

[115] Chung J, Park J, Lai ZW, Lambert TJ, Richards RC, Zhang J (2023). The Troyer syndrome protein spartin mediates selective autophagy of lipid droplets. Nat Cell Biol.

[116] Tabe Y, Konopleva M, Andreeff M (2020). Fatty acid metabolism, bone marrow adipocytes, and AML. Front Oncol.

[117] Komiya K, Uchida T, Ueno T, Koike M, Abe H, Hirose T (2010). Free fatty acids stimulate autophagy in pancreatic β-cells via JNK pathway. Biochem Biophys Res Commun.

[118] Guo W, Zhong W, Hao L, Sun X, Zhou Z (2021). Activation of mTORC1 by free fatty Acids suppresses LAMP2 and autophagy function via ER stress in alcohol-related liver disease. Cells.

[119] Kim KH, Lee MS (2014). Autophagy-a key player in cellular and body metabolism. Nat Rev Endocrinol.

[120] Tomaipitinca L, Mandatori S, Mancinelli R, Giulitti F, Petrungaro S, Moresi V (2019). The role of autophagy in liver epithelial cells and its impact on systemic homeostasis. Nutrients.

[121] Zhang X, Evans TD, Jeong SJ, Razani B (2018). Classical and alternative roles for autophagy in lipid metabolism. Curr Opin Lipidol.

[122] Humbert M, Seiler K, Mosimann S, Rentsch V, Sharma K, Pandey AV (2021). Reducing FASN expression sensitizes acute myeloid leukemia cells to differentiation therapy. Cell Death Differ.

[123] Huang N, Ren J, Deng X, Bao Q, Huang G, Zhi S (2025). Endothelial F3-mediated autolysosome and lipid metabolism promote resistance to anti-VEGFA therapy in metastatic colorectal cancer. Autophagy.

[124] Tang M, Dong X, Xiao L, Tan Z, Luo X, Yang L (2022). CPT1A-mediated fatty acid oxidation promotes cell proliferation via nucleoside metabolism in nasopharyngeal carcinoma. Cell Death Dis.

[125] Jin HR, Wang J, Wang ZJ, Xi MJ, Xia BH, Deng K (2023). Lipid metabolic reprogramming in tumor microenvironment: from mechanisms to therapeutics. J Hematol Oncol.

[126] Ye H, Adane B, Khan N, Sullivan T, Minhajuddin M, Gasparetto M (2016). Leukemic stem cells evade chemotherapy by metabolic adaptation to an adipose tissue niche. Cell Stem Cell.

[127] Tabe Y, Yamamoto S, Saitoh K, Sekihara K, Monma N, Ikeo K (2017). Bone marrow adipocytes facilitate fatty acid oxidation activating AMPK and a transcriptional network supporting survival of acute monocytic leukemia cells. Cancer Res.

[128] Maan M, Peters JM, Dutta M, Patterson AD (2018). Lipid metabolism and lipophagy in cancer. Biochem Biophys Res Commun.

[129] Kaushik S, Cuervo AM (2015). Degradation of lipid droplet-associated proteins by chaperone-mediated autophagy facilitates lipolysis. Nat Cell Biol.

[130] Patanè GT, Putaggio S, Tellone E, Barreca D, Ficarra S, Maffei C (2023). Ferroptosis: emerging role in diseases and potential implication of bioactive compounds. Int J Mol Sci.

[131] Hanahan D, Weinberg RA (2011). Hallmarks of cancer: the next generation. Cell.

[132] Bensaad K, Favaro E, Lewis CA, Peck B, Lord S, Collins JM (2014). Fatty acid uptake and lipid storage induced by HIF-1α contribute to cell growth and survival after hypoxia-reoxygenation. Cell Rep.

[133] Song BH, Son SY, Kim HK, Ha TW, Im JS, Ryu A (2020). Profiling of metabolic differences between hematopoietic stem cells and acute/chronic myeloid leukemia. metabolites.

[134] Vettore L, Westbrook RL, Tennant DA (2020). New aspects of amino acid metabolism in cancer. Br J Cancer.

[135] Jin J, Byun JK, Choi YK, Park KG (2023). Targeting glutamine metabolism as a therapeutic strategy for cancer. Exp Mol Med.

[136] Li S, Zeng H, Fan J, Wang F, Xu C, Li Y (2023). Glutamine metabolism in breast cancer and possible therapeutic targets. Biochem Pharmacol.

[137] Douiev L, Miller C, Ruppo S, Benyamini H, Abu-Libdeh B, Saada A (2021). Upregulation of COX4-2 via HIF-1α in mitochondrial COX4-1 deficiency. Cells.

[138] Zhang T, Liu Q, Gao W, Sehgal SA, Wu H (2022). The multifaceted regulation of mitophagy by endogenous metabolites. Autophagy.

[139] Wang Y, Bai C, Ruan Y, Liu M, Chu Q, Qiu L (2019). Coordinative metabolism of glutamine carbon and nitrogen in proliferating cancer cells under hypoxia. Nat Commun.

[140] Ko YH, Lin Z, Flomenberg N, Pestell RG, Howell A, Sotgia F (2011). Glutamine fuels a vicious cycle of autophagy in the tumor stroma and oxidative mitochondrial metabolism in epithelial cancer cells: implications for preventing chemotherapy resistance. Cancer Biol Ther.

[141] Nie C, He T, Zhang W, Zhang G, Ma X (2018). Branched chain amino acids: beyond nutrition metabolism. Int J Mol Sci.

[142] Wang T, Hu Q, Li B, Fan G, Jing D, Xu J (2024). Transcription factor EB reprograms branched-chain amino acid metabolism and promotes pancreatic cancer progression via transcriptional regulation of BCAT1. Cell Prolif.

[143] Mei W, Wei M, Tang C, Li W, Ye B, Xin S (2025). BCAT2 binding to PCBP1 regulates the PI3K/AKT signaling pathway to inhibit autophagy-related apoptosis and ferroptosis in prostate cancer. Cell Death Dis.

[144] Sivanand S, Vander Heiden MG (2020). Emerging roles for branched-chain amino acid metabolism in cancer. Cancer Cell.

[145] Perveen R, Ozaki I, Takahashi H, Manirujjaman M, Kuwashiro T, Matsuhashi S (2025). BCAA (Branched-Chain Amino Acids) inhibiting the autophagy system via the activation of mTORC1, thereby upregulating the tumor suppressor PDCD4 in Huh7 hepatoma cells. Cells.

[146] B'Chir W, Maurin AC, Carraro V, Averous J, Jousse C, Muranishi Y (2013). The eIF2α/ATF4 pathway is essential for stress-induced autophagy gene expression. Nucleic Acids Res.

[147] Brown KK (2021). AMPK CA(R)Sts a new light on amino acid sensing. Embo j.

[148] Kim DH (2024). Contrasting views on the role of AMPK in autophagy. Bioessays.

[149] Dossou AS, Basu A (2019). The emerging roles of mTORC1 in macromanaging autophagy. Cancers (Basel).

[150] Bettedi L, Zhang Y, Yang S, Lilly MA (2024). Unveiling GATOR2 function: Novel insights from drosophila research. Cells.

[151] Yao Y, Jones E, Inoki K (2017). Lysosomal Regulation of mTORC1 by amino acids in mammalian cells. Biomolecules.

[152] Kim J, Guan KL (2019). mTOR as a central hub of nutrient signalling and cell growth. Nat Cell Biol.

[153] Zhang D, Song S, Lin J, Ye T, Yang X, Jiang Q (2025). Glutamine binds HSC70 to transduce signals inhibiting IFN-β-mediated immunogenic cell death. Dev Cell.

[154] Jin L, Chun J, Pan C, Alesi GN, Li D, Magliocca KR (2017). Phosphorylation-mediated activation of LDHA promotes cancer cell invasion and tumour metastasis. Oncogene.

[155] Zhao Y, Mudge MC, Soll JM, Rodrigues RB, Byrum AK, Schwarzkopf EA (2018). OTUD4 is a phospho-activated K63 deubiquitinase that regulates MyD88-dependent signaling. Mol Cell.

[156] Kastenhuber ER, Lowe SW (2017). Putting p53 in Context. Cell.

[157] Mrakovcic M, Fröhlich LF (2018). p53-Mediated molecular control of autophagy in tumor cells. Biomolecules.

[158] Hu W, Zhang C, Wu R, Sun Y, Levine A, Feng Z (2010). Glutaminase 2, a novel p53 target gene regulating energy metabolism and antioxidant function. Proc Natl Acad Sci U S A.

[159] Shi DD, Savani MR, Abdullah KG, McBrayer SK (2023). Emerging roles of nucleotide metabolism in cancer. Trends Cancer.

[160] Mullen NJ, Singh PK (2023). Nucleotide metabolism: a pan-cancer metabolic dependency. Nat Rev Cancer.

[161] Guo JY, Teng X, Laddha SV, Ma S, Van Nostrand SC, Yang Y (2016). Autophagy provides metabolic substrates to maintain energy charge and nucleotide pools in Ras-driven lung cancer cells. Genes Dev.

[162] Dufresne S, Kuna RS, Wong K, Komarla A, Rock A, Rosada-Encarnación J Leveraging autophagy and pyrimidine metabolism to target pancreatic cancer. bioRxiv. 2025: 656904.

[163] Chen T, Zheng L, Luo P, Zou J, Li W, Chen Q (2024). Crosstalk between m6A modification and autophagy in cancer. Cell Biosci.

[164] Chen X, Wang J, Tahir M, Zhang F, Ran Y, Liu Z (2021). Current insights into the implications of m6A RNA methylation and autophagy interaction in human diseases. Cell Biosci.

[165] Chen Y, Wang J, Xu D, Xiang Z, Ding J, Yang X (2021). m(6)A mRNA methylation regulates testosterone synthesis through modulating autophagy in Leydig cells. Autophagy.

[166] Tian J, Zhu Y, Rao M, Cai Y, Lu Z, Zou D (2020). N(6)-methyladenosine mRNA methylation of PIK3CB regulates AKT signalling to promote PTEN-deficient pancreatic cancer progression. Gut.

[167] Xia L, Xing Y, Ye X, Wu Y, Yang Y, Yin Z (2025). TRIM21-driven K63-linked ubiquitination of RBM38c, as a novel interactor of BECN1, contributes to DNA damage-induced autophagy. Cell Death Differ.

[168] Chentunarayan Singh N, Yadav N, Sharma RK, Gupta P, Sarkar J, Mitra K (2026). Oxidative stress-mediated DNA damage promotes selective degradation of nuclear components via noncanonical autophagy in triple-negative breast cancer cells. Free Radic Biol Med.

[169] Sun Z, Liu L, Liang H, Zhang L (2024). Nicotinamide mononucleotide induces autophagy and ferroptosis via AMPK/mTOR pathway in hepatocellular carcinoma. Mol Carcinog.

[170] Dong Y, Zhang X (2024). Targeting cellular mitophagy as a strategy for human cancers. Front Cell Dev Biol.

[171] Chu T, Huang Z, Ma W (2025). Mitophagy: A double-edged sword in tumor cell death regulation and therapeutic response. Biochem Biophys Res Commun.

[172] Rui K, Qiu J, Li M, Huang J, Wang T, Yin K (2026). Crosstalk between mitophagy and breast cancer: mechanisms of action and clinical applications. J Cancer Res Clin Oncol.

[173] Zhang Y, Shen X, Shen Y, Wang C, Yu C, Han J (2026). Cytosolic acetyl-coenzyme A is a signalling metabolite to control mitophagy. Nature.

[174] Lu D, Li Y, Niu X, Sun J, Zhan W, Shi Y (2024). STAT2/SLC27A3/PINK1-mediated mitophagy remodeling lipid metabolism contributes to pazopanib resistance in clear cell renal cell carcinoma. Research (Wash D C).

[175] Luo P, An Y, He J, Xing X, Zhang Q, Liu X (2024). Icaritin with autophagy/mitophagy inhibitors synergistically enhances anticancer efficacy and apoptotic effects through PINK1/Parkin-mediated mitophagy in hepatocellular carcinoma. Cancer Lett.

[176] Debnath J, Gammoh N, Ryan KM (2023). Autophagy and autophagy-related pathways in cancer. Nat Rev Mol Cell Biol.

[177] Galluzzi L, Green DR (2019). Autophagy-independent functions of the autophagy machinery. cell.

[178] Kang R, Zeh H, Lotze M, Tang D (2020). The multifaceted effects of autophagy on the tumor microenvironment. Adv Exp Med Biol.

[179] Sikder S, Mondal A, Das C, Kundu TK (2022). Autophagy in cancer: A metabolic perspective. subcell biochem.

[180] Mulcahy Levy JM, Thorburn A (2020). Autophagy in cancer: moving from understanding mechanism to improving therapy responses in patients. Cell Death Differ.

[181] El Hout M, Cosialls E, Mehrpour M, Hamaï A (2020). Crosstalk between autophagy and metabolic regulation of cancer stem cells. Mol Cancer.

[182] Wong KKL, Verheyen EM (2021). Metabolic reprogramming in cancer: mechanistic insights from Drosophila. Dis Model Mech.

[183] Zhao H, Li Y (2021). Cancer metabolism and intervention therapy. Mol Biomed.

[184] Trajkovic-Arsic M, Subramani E (2023). Is metabolism the magic bullet for targeted cancer therapy?. BMC Cancer.

[185] Wang YH, Israelsen WJ, Lee D, Yu VWC, Jeanson NT, Clish CB (2014). Cell-state-specific metabolic dependency in hematopoiesis and leukemogenesis. Cell.

[186] Chen S, Tao Y, Wang Q, Ren J, Jing Y, Huang J (2023). Glucose induced-AKT/mTOR activation accelerates glycolysis and promotes cell survival in acute myeloid leukemia. Leuk Res.

[187] Huldani H, Malviya J, Rodrigues P, Hjazi A, Deorari MM, Al-Hetty H (2024). Discovering the strength of immunometabolism in cancer therapy: Employing metabolic pathways to enhance immune responses. Cell Biochem Funct.

[188] Latham BD, Geffert RM, Jackson KD (2024). Kinase inhibitors FDA approved 2018-2023: drug targets, metabolic pathways, and drug-induced toxicities. Drug Metab Dispos.

[189] Tufail M, Jiang CH, Li N (2024). Altered metabolism in cancer: insights into energy pathways and therapeutic targets. Mol Cancer.

[190] Pajak B, Siwiak E, Sołtyka M, Priebe A, Zieliński R, Fokt I (2019). 2-Deoxy-d-glucose and its analogs: From diagnostic to therapeutic agents. Int J Mol Sci.

[191] Wang Q, Liang B, Shirwany NA, Zou MH (2011). 2-Deoxy-D-glucose treatment of endothelial cells induces autophagy by reactive oxygen species-mediated activation of the AMP-activated protein kinase. PLoS One.

[192] Li J, Tong D, Lin J (2022). Current status of cancer starvation therapy. Zhejiang Da Xue Xue Bao Yi Xue Ban.

[193] Yang B, Ding L, Chen Y, Shi J (2020). Augmenting tumor-starvation therapy by cancer cell autophagy inhibition. Adv Sci (Weinh).

[194] Li J, Pan J, Liu Y, Luo X, Yang C, Xiao W (2022). 3-Bromopyruvic acid regulates glucose metabolism by targeting the c-Myc/TXNIP axis and induces mitochondria-mediated apoptosis in TNBC cells. Exp Ther Med.

[195] Zhao B, Aggarwal A, Marshall JA, Barletta JA, Kijewski MF, Lorch JH (2022). Glycolytic inhibition with 3-bromopyruvate suppresses tumor growth and improves survival in a murine model of anaplastic thyroid cancer. Surgery.

[196] Li MY, Shen HH, Cao XY, Gao XX, Xu FY, Ha SY (2024). Targeting a mTOR/autophagy axis: a double-edged sword of rapamycin in spontaneous miscarriage. Biomed Pharmacother.

[197] Gan L, Ren Y, Lu J, Ma J, Shen X, Zhuang Z (2020). Synergistic effect of 3-bromopyruvate in combination with rapamycin impacted neuroblastoma metabolism by inhibiting autophagy. Onco Targets Ther.

[198] Zhao H, Swanson KD, Zheng B (2021). Therapeutic repurposing of biguanides in cancer. trends cancer.

[199] Cunha Júnior AD, Bragagnoli AC, Costa FO, Carvalheira JBC (2021). Repurposing metformin for the treatment of gastrointestinal cancer. World J Gastroenterol.

[200] LaMoia TE, Shulman GI (2021). Cellular and molecular mechanisms of metformin action. Endocr Rev.

[201] Rena G, Hardie DG, Pearson ER (2017). The mechanisms of action of metformin. Diabetologia.

[202] Kamarudin MNA, Sarker MMR, Zhou JR, Parhar I (2019). Metformin in colorectal cancer: molecular mechanism, preclinical and clinical aspects. J Exp Clin Cancer Res.

[203] De A, Kuppusamy G (2020). Metformin in breast cancer: preclinical and clinical evidence. Curr Probl Cancer.

[204] Zell JA, McLaren CE, Morgan TR, Lawson MJ, Rezk S, Albers CG (2020). A phase IIa trial of metformin for colorectal cancer risk reduction among individuals with history of colorectal adenomas and elevated body mass index. Cancer Prev Res (Phila).

[205] Liu Q, Xu X, Zhao M, Wei Z, Li X, Zhang X (2015). Berberine induces senescence of human glioblastoma cells by downregulating the EGFR-MEK-ERK signaling pathway. Mol Cancer Ther.

[206] Lu N, Tong Z, Zhang M, Lu L, Cao H (2015). [Effect and mechanism of EGFR expression in macrophages on the anti-cancer effect of berberine on colorectal cancer]. Zhonghua Zhong Liu Za Zhi.

[207] Lu JJ, Fu L, Tang Z, Zhang C, Qin L, Wang J (2016). Melatonin inhibits AP-2β/hTERT, NF-κB/COX-2 and Akt/ERK and activates caspase/Cyto C signaling to enhance the antitumor activity of berberine in lung cancer cells. Oncotarget.

[208] Tian Y, Zhao L, Wang Y, Zhang H, Xu D, Zhao X (2016). Berberine inhibits androgen synthesis by interaction with aldo-keto reductase 1C3 in 22Rv1 prostate cancer cells. Asian J Androl.

[209] Chen Q, Qin R, Fang Y, Li H (2015). Berberine sensitizes human ovarian cancer cells to cisplatin through miR-93/PTEN/Akt signaling pathway. Cell Physiol Biochem.

[210] Bettum IJ, Gorad SS, Barkovskaya A, Pettersen S, Moestue SA, Vasiliauskaite K (2015). Metabolic reprogramming supports the invasive phenotype in malignant melanoma. Cancer Lett.

[211] Wang J, Qi Q, Feng Z, Zhang X, Huang B, Chen A (2016). Berberine induces autophagy in glioblastoma by targeting the AMPK/mTOR/ULK1-pathway. Oncotarget.

[212] Park JH, Kundu A, Lee SH, Jiang C, Lee SH, Kim YS (2021). Specific pyruvate kinase M2 inhibitor, compound 3K, induces autophagic cell death through disruption of the glycolysis pathway in ovarian cancer cells. Int J Biol Sci.

[213] Zhu S, Guo Y, Zhang X, Liu H, Yin M, Chen X (2021). Pyruvate kinase M2 (PKM2) in cancer and cancer therapeutics. Cancer Lett.

[214] Pereira CV, Duarte M, Silva P, Bento da Silva A, Duarte CMM, Cifuentes A (2019). Polymethoxylated flavones target cancer stemness and improve the antiproliferative effect of 5-Fluorouracil in a 3D cell model of colorectal cancer. Nutrients.

[215] Wang L, Wang J, Fang L, Zheng Z, Zhi D, Wang S (2014). Anticancer activities of citrus peel polymethoxyflavones related to angiogenesis and others. Biomed Res Int.

[216] Yin Y, Wu YU, Huang H, Duan Y, Yuan Z, Cao L (2024). The superiority of PMFs on reversing drug resistance of colon cancer and the effect on aerobic glycolysis-ROS-autophagy signaling axis. Oncol Res.

[217] Tanner LB, Goglia AG, Wei MH, Sehgal T, Parsons LR, Park JO (2018). Four key steps control glycolytic flux in mammalian cells. Cell Syst.

[218] Heydarzadeh S, Moshtaghie AA, Daneshpoor M, Hedayati M (2020). Regulators of glucose uptake in thyroid cancer cell lines. Cell Commun Signal.

[219] Chan DA, Sutphin PD, Nguyen P, Turcotte S, Lai EW, Banh A (2011). Targeting GLUT1 and the Warburg effect in renal cell carcinoma by chemical synthetic lethality. Sci Transl Med.

[220] Siebeneicher H, Cleve A, Rehwinkel H, Neuhaus R, Heisler I, Müller T (2016). Identification and optimization of the first highly selective GLUT1 inhibitor BAY-876. ChemMedChem.

[221] Kuo CY, Hsu YC, Chen MJ, Lin CH, Li YS, Cheng SP (2025). Glucose transporter 1 inhibitors induce autophagy and synergize with lenvatinib in thyroid cancer cells. Head Neck.

[222] Hung HC, Li LC, Guh JH, Kung FL, Hsu LC (2022). Discovery of new glucose uptake inhibitors as potential anticancer agents by non-radioactive cell-based assays. Molecules.

[223] Sawayama H, Ogata Y, Ishimoto T, Mima K, Hiyoshi Y, Iwatsuki M (2019). Glucose transporter 1 regulates the proliferation and cisplatin sensitivity of esophageal cancer. Cancer Sci.

[224] Bahremani M, Rashtchizadeh N, Sabzichi M, Vatankhah AM, Danaiyan S, Poursistany H (2023). Enhanced chemotherapeutic efficacy of docetaxel in human lung cancer cell line via GLUT1 inhibitor. J Biochem Mol Toxicol.

[225] Liu J, Xia H, Kim M, Xu L, Li Y, Zhang L (2011). Beclin1 controls the levels of p53 by regulating the deubiquitination activity of USP10 and USP13. Cell.

[226] Kunimasa K, Ikeda-Ishikawa C, Tani Y, Tsukahara S, Sakurai J, Okamoto Y (2022). Spautin-1 inhibits mitochondrial complex I and leads to suppression of the unfolded protein response and cell survival during glucose starvation. Sci Rep.

[227] Li TY, Sun Y, Liang Y, Liu Q, Shi Y, Zhang CS (2016). ULK1/2 constitute a bifurcate node controlling glucose metabolic fluxes in addition to autophagy. Mol Cell.

[228] Li Z, Lu Y, Yuan Z, He J, Li H, Yang Q (2025). Computational identification of potential UNC-51-like kinase 1 inhibitors by an integrated structure-based and molecular dynamics-guided approach. Int J Biol Macromol.

[229] Egan DF, Chun MG, Vamos M, Zou H, Rong J, Miller CJ (2015). Small molecule inhibition of the autophagy kinase ULK1 and identification of ULK1 substrates. Mol Cell.

[230] Tang F, Hu P, Yang Z, Xue C, Gong J, Sun S (2017). SBI0206965, a novel inhibitor of Ulk1, suppresses non-small cell lung cancer cell growth by modulating both autophagy and apoptosis pathways. Oncol Rep.

[231] TeSlaa T, Ralser M, Fan J, Rabinowitz JD (2023). The pentose phosphate pathway in health and disease. Nat Metab.

[232] Lu J, Zhu L, Zheng LP, Cui Q, Zhu HH, Zhao H (2018). Overexpression of ULK1 represents a potential diagnostic marker for clear cell renal carcinoma and the antitumor effects of SBI-0206965. EBioMedicine.

[233] Donohue E, Thomas A, Maurer N, Manisali I, Zeisser-Labouebe M, Zisman N (2013). The autophagy inhibitor verteporfin moderately enhances the antitumor activity of gemcitabine in a pancreatic ductal adenocarcinoma model. J Cancer.

[234] Kumar D, Shankar S, Srivastava RK (2014). Rottlerin induces autophagy and apoptosis in prostate cancer stem cells via PI3K/Akt/mTOR signaling pathway. Cancer Lett.

[235] Torricelli C, Daveri E, Salvadori S, Valacchi G, Ietta F, Muscettola M (2015). Phosphorylation-independent mTORC1 inhibition by the autophagy inducer Rottlerin. Cancer Lett.

[236] Yan LS, Zhang SF, Luo G, Cheng BC, Zhang C, Wang YW (2022). Schisandrin B mitigates hepatic steatosis and promotes fatty acid oxidation by inducing autophagy through AMPK/mTOR signaling pathway. Metabolism.

[237] Li Q, Wang Z, Xie Y, Hu H (2020). Antitumor activity and mechanism of costunolide and dehydrocostus lactone: Two natural sesquiterpene lactones from the Asteraceae family. Biomed Pharmacother.

[238] Bian X, Liu R, Meng Y, Xing D, Xu D, Lu Z (2021). Lipid metabolism and cancer. J Exp Med.

[239] Chen Y, Shen J, Yuan M, Li H, Li Y, Zheng S (2025). Dehydrocostus lactone suppresses gastric cancer progression by targeting ACLY to inhibit fatty acid synthesis and autophagic flux. J Adv Res.

[240] Myers RW, Guan HP, Ehrhart J, Petrov A, Prahalada S, Tozzo E (2017). Systemic pan-AMPK activator MK-8722 improves glucose homeostasis but induces cardiac hypertrophy. Science.

[241] Pang ZD, Wang Y, Song Z, She G, Ma XZ, Sun X (2021). AMPK upregulates K(Ca)2.3 channels and ameliorates endothelial dysfunction in diet-induced obese mice. Biochem Pharmacol.

[242] Zhou X, Muise ES, Haimbach R, Sebhat IK, Zhu Y, Liu F (2019). PAN-AMPK activation improves renal function in a rat model of progressive diabetic nephropathy. J Pharmacol Exp Ther.

[243] Wang L, Zhu H, Shi Z, Chen B, Huang H, Lin G (2024). MK8722 initiates early-stage autophagy while inhibiting late-stage autophagy via FASN-dependent reprogramming of lipid metabolism. Theranostics.

[244] Alistar A, Morris BB, Desnoyer R, Klepin HD, Hosseinzadeh K, Clark C (2017). Safety and tolerability of the first-in-class agent CPI-613 in combination with modified FOLFIRINOX in patients with metastatic pancreatic cancer: a single-centre, open-label, dose-escalation, phase 1 trial. Lancet Oncol.

[245] Chen L, Duan Y, Wei H, Ning H, Bi C, Zhao Y (2019). Acetyl-CoA carboxylase (ACC) as a therapeutic target for metabolic syndrome and recent developments in ACC1/2 inhibitors. Expert Opin Investig Drugs.

[246] Gao L, Xu Z, Huang Z, Tang Y, Yang D, Huang J (2020). CPI-613 rewires lipid metabolism to enhance pancreatic cancer apoptosis via the AMPK-ACC signaling. J Exp Clin Cancer Res.

[247] Zhang L, Fu L, Zhang S, Zhang J, Zhao Y, Zheng Y (2017). Discovery of a small molecule targeting ULK1-modulated cell death of triple negative breast cancer in vitro and in vivo. Chem Sci.

[248] An M, Ryu DR, Won Park J, Ha Choi J, Park EM, Eun Lee K (2017). ULK1 prevents cardiac dysfunction in obesity through autophagy-meditated regulation of lipid metabolism. Cardiovasc Res.

[249] Walther TC, Chung J, Farese RV Jr (2017). Lipid droplet biogenesis. Annu Rev Cell Dev Biol.

[250] Zhang C, Liu P (2017). The lipid droplet: A conserved cellular organelle. Protein Cell.

[251] Mondal S, Roy D, Sarkar Bhattacharya S, Jin L, Jung D, Zhang S (2019). Therapeutic targeting of PFKFB3 with a novel glycolytic inhibitor PFK158 promotes lipophagy and chemosensitivity in gynecologic cancers. Int J Cancer.

[252] S SK, S NS, Devaraj VR, Premalatha CS, Pallavi VR, Sagar BC (2022). Metabolic reprogramming and lipophagy mediates survival of ascites derived metastatic ovarian cancer cells. Asian Pac J Cancer Prev.

[253] Holubarsch CJ, Rohrbach M, Karrasch M, Boehm E, Polonski L, Ponikowski P (2007). A double-blind randomized multicentre clinical trial to evaluate the efficacy and safety of two doses of etomoxir in comparison with placebo in patients with moderate congestive heart failure: the ERGO (etomoxir for the recovery of glucose oxidation) study. Clin Sci (Lond).

[254] Jafari N, Drury J, Morris AJ, Onono FO, Stevens PD, Gao T (2019). De novo fatty acid synthesis-driven sphingolipid metabolism promotes metastatic potential of colorectal cancer. Mol Cancer Res.

[255] Ye J, DeBose-Boyd RA (2011). Regulation of cholesterol and fatty acid synthesis. Cold Spring Harb Perspect Biol.

[256] Varoni EM, Lo Faro AF, Sharifi-Rad J, Iriti M (2016). Anticancer molecular mechanisms of resveratrol. Front Nutr.

[257] Marques FZ, Markus MA, Morris BJ (2009). Resveratrol: cellular actions of a potent natural chemical that confers a diversity of health benefits. Int J Biochem Cell Biol.

[258] Zhu Y, He W, Gao X, Li B, Mei C, Xu R (2015). Resveratrol overcomes gefitinib resistance by increasing the intracellular gefitinib concentration and triggering apoptosis, autophagy and senescence in PC9/G NSCLC cells. Sci Rep.

[259] Selvaraj S, Sun Y, Sukumaran P, Singh BB (2016). Resveratrol activates autophagic cell death in prostate cancer cells via downregulation of STIM1 and the mTOR pathway. Mol Carcinog.

[260] Fukuda M, Ogasawara Y, Hayashi H, Inoue K, Sakashita H (2022). Resveratrol inhibits proliferation and induces autophagy by blocking SREBP1 expression in oral cancer cells. Molecules.

[261] Hensley CT, Wasti AT, DeBerardinis RJ (2013). Glutamine and cancer: cell biology, physiology, and clinical opportunities. J Clin Invest.

[262] Schulte ML, Fu A, Zhao P, Li J, Geng L, Smith ST (2018). Pharmacological blockade of ASCT2-dependent glutamine transport leads to antitumor efficacy in preclinical models. Nat Med.

[263] Xiang Y, Stine ZE, Xia J, Lu Y, O'Connor RS, Altman BJ (2015). Targeted inhibition of tumor-specific glutaminase diminishes cell-autonomous tumorigenesis. J Clin Invest.

[264] Beier AK, Ebersbach C, Siciliano T, Scholze J, Hofmann J, Hönscheid P (2024). Targeting the glutamine metabolism to suppress cell proliferation in mesenchymal docetaxel-resistant prostate cancer. Oncogene.

[265] Nan D, Yao W, Huang L, Liu R, Chen X, Xia W (2025). Glutamine and cancer: metabolism, immune microenvironment, and therapeutic targets. Cell Commun Signal.

[266] Lampa M, Arlt H, He T, Ospina B, Reeves J, Zhang B (2017). Glutaminase is essential for the growth of triple-negative breast cancer cells with a deregulated glutamine metabolism pathway and its suppression synergizes with mTOR inhibition. PLoS One.

[267] Pachow D, Wick W, Gutmann DH, Mawrin C (2015). The mTOR signaling pathway as a treatment target for intracranial neoplasms. Neuro Oncol.

[268] Yamashita AS, da Costa Rosa M, Stumpo V, Rais R, Slusher BS, Riggins GJ (2021). The glutamine antagonist prodrug JHU-083 slows malignant glioma growth and disrupts mTOR signaling. Neurooncol Adv.

[269] Oh MH, Sun IH, Zhao L, Leone RD, Sun IM, Xu W (2020). Targeting glutamine metabolism enhances tumor-specific immunity by modulating suppressive myeloid cells. J Clin Invest.

[270] Ohshima Y, Kaira K, Yamaguchi A, Oriuchi N, Tominaga H, Nagamori S (2016). Efficacy of system l amino acid transporter 1 inhibition as a therapeutic target in esophageal squamous cell carcinoma. Cancer Sci.

[271] Takeuchi K, Ogata S, Nakanishi K, Ozeki Y, Hiroi S, Tominaga S (2010). LAT1 expression in non-small-cell lung carcinomas: analyses by semiquantitative reverse transcription-PCR (237 cases) and immunohistochemistry (295 cases). Lung Cancer.

[272] Altan B, Kaira K, Watanabe A, Kubo N, Bao P, Dolgormaa G (2018). Relationship between LAT1 expression and resistance to chemotherapy in pancreatic ductal adenocarcinoma. Cancer Chemother Pharmacol.

[273] Wu T, Yu Z, Dai J, Li J, Ning F, Liu X (2024). JPH203 alleviates peritoneal fibrosis via inhibition of amino acid-mediated mTORC1 signaling. Biochem Biophys Res Commun.

[274] Bo T, Kobayashi S, Inanami O, Fujii J, Nakajima O, Ito T (2021). LAT1 inhibitor JPH203 sensitizes cancer cells to radiation by enhancing radiation-induced cellular senescence. Transl Oncol.

[275] Kongpracha P, Nagamori S, Wiriyasermkul P, Tanaka Y, Kaneda K, Okuda S (2017). Structure-activity relationship of a novel series of inhibitors for cancer type transporter L-type amino acid transporter 1 (LAT1). J Pharmacol Sci.

[276] Zhang D, Wang Y, Yu P, Sun J, Li J, Hu Y (2025). Scutellarein inhibits lung cancer growth by inducing cell apoptosis and inhibiting glutamine metabolic pathway. J Ethnopharmacol.

[277] Li J, Zhang D, Wang S, Yu P, Sun J, Zhang Y (2025). Baicalein induces apoptosis by inhibiting the glutamine-mTOR metabolic pathway in lung cancer. J Adv Res.

[278] Yang Y, He P, Hou Y, Liu Z, Zhang X, Li N (2022). Osmundacetone modulates mitochondrial metabolism in non-small cell lung cancer cells by hijacking the glutamine/glutamate/α-KG metabolic axis. Phytomedicine.

[279] Liu R, Yang G, Bao M, Zhou Z, Mao X, Liu W (2022). STAMBPL1 promotes breast cancer cell resistance to cisplatin partially by stabilizing MKP-1 expression. Oncogene.

[280] Qi J, Zulfiker AHM, Li C, Good D, Wei MQ (2018). The development of toad toxins as potential therapeutic agents. Toxins (Basel).

[281] Zheng P, Xu D, Cai Y, Zhu L, Xiao Q, Peng W (2024). A multi-omic analysis reveals that Gamabufotalin exerts anti-hepatocellular carcinoma effects by regulating amino acid metabolism through targeting STAMBPL1. Phytomedicine.

[282] Holbert CE, Cullen MT, Casero RA Jr, Stewart TM (2022). Polyamines in cancer: integrating organismal metabolism and antitumour immunity. Nat Rev Cancer.

[283] Brune MM, Savic Prince S, Vlajnic T, Chijioke O, Roma L, König D (2024). MTAP as an emerging biomarker in thoracic malignancies. Lung Cancer.

[284] Tang B, Lee HO, An SS, Cai KQ, Kruger WD (2018). Specific targeting of MTAP-deleted tumors with a combination of 2'-fluoroadenine and 5'-methylthioadenosine. Cancer Res.

[285] Patro CPK, Biswas N, Pingle SC, Lin F, Anekoji M, Jones LD (2022). MTAP loss: a possible therapeutic approach for glioblastoma. J Transl Med.

[286] Coorey NV, Tollestrup I, Bircham PW, Sheridan JP, Evans GB, Schramm VL (2025). The anti-cancer transition-state inhibitor MTDIA inhibits human MTAP, inducing autophagy in humanized yeast. Dis Model Mech.

[287] Vykoukal J, Chen Y, Zuo M, Ballarò R, Hong MJ, Krishna H (2025). Vesicle-mediated mitochondrial clearance presents an actionable metabolic vulnerability in triple-negative breast cancer. Cell Rep Med.

[288] Chang H, Hou J, Hu X, Hou N, Xu M, Du Y (2025). Sanggenol L Enhances temozolomide drug sensitivity by inhibiting mitophagy and inducing apoptosis through the regulation of the TRIM16-OPTN axis in glioblastoma. Adv Sci (Weinh).

[289] Huang JL, Wu LM, Wu SQ, Yuan FY, Weng HZ, Huang D (2026). A small molecule targets LIC1 to suppress lung tumor growth by inducing autophagy. Nat Chem Biol.

[290] Li XJ, Huang KX, Wang KX, Liu R, Wang DC, Liang YR (2025). Design, synthesis, and antitumor activity of novel thioheterocyclic nucleoside derivatives by suppressing the c-MYC pathway. Acta Pharm Sin B.

[291] Haas NB, Appleman LJ, Stein M, Redlinger M, Wilks M, Xu X (2019). Autophagy inhibition to augment mTOR inhibition: a phase I/II trial of everolimus and hydroxychloroquine in patients with previously treated renal cell carcinoma. Clin Cancer Res.

[292] Tang H, Ge Y, You T, Li X, Wang Y, Cheng Y (2023). A real-world analysis of trametinib in combination with hydroxychloroquine or CDK4/6 inhibitor as third- or later-line therapy in metastatic pancreatic adenocarcinoma. BMC Cancer.

[293] Witte D, Pretzell I, Reissig TM, Stein A, Velthaus JL, Alig A (2024). Trametinib in combination with hydroxychloroquine or palbociclib in advanced metastatic pancreatic cancer: data from a retrospective, multicentric cohort (AIO AIO-TF/PAK-0123). J Cancer Res Clin Oncol.

[294] Zamanian MY, Golmohammadi M, Yumashev A, Hjazi A, Toama MA, AbdRabou MA (2024). Effects of metformin on cancers in experimental and clinical studies: Focusing on autophagy and AMPK/mTOR signaling pathways. Cell Biochem Funct.

[295] Chomanicova N, Gazova A, Adamickova A, Valaskova S, Kyselovic J (2021). The role of AMPK/mTOR signaling pathway in anticancer activity of metformin. Physiol Res.

[296] Hadad SM, Coates P, Jordan LB, Dowling RJ, Chang MC, Done SJ (2015). Evidence for biological effects of metformin in operable breast cancer: biomarker analysis in a pre-operative window of opportunity randomized trial. Breast Cancer Res Treat.

[297] Lee CH, Motzer R, Emamekhoo H, Matrana M, Percent I, Hsieh JJ (2022). Telaglenastat plus everolimus in advanced renal cell carcinoma: A randomized, double-blinded, placebo-controlled, phase II ENTRATA trial. Clin Cancer Res.

[298] Tannir NM, Agarwal N, Porta C, Lawrence NJ, Motzer R, McGregor B (2022). Efficacy and safety of telaglenastat plus cabozantinib vs placebo plus cabozantinib in patients with advanced renal cell carcinoma: The CANTATA randomized clinical trial. JAMA Oncol.

[299] Raczka AM, Reynolds PA (2019). Glutaminase inhibition in renal cell carcinoma therapy. Cancer Drug Resist.

[300] Yu YS, Kim IS, Baek SH (2025). Decoding the dual role of autophagy in cancer through transcriptional and epigenetic regulation. FEBS Lett.

[301] Qu Z, Pen X, Liu Y, Pen X, Lu X, Luo S (2025). Autophagy in colorectal cancer: Current understanding of mechanisms and future therapeutic directions. Biomed Pharmacother.

